# Analytical validation of a multiplexed peptide microarray for multi-antigen Epstein–Barr virus serological profiling across diverse clinical specimens

**DOI:** 10.3389/fimmu.2026.1919983

**Published:** 2026-09-03

**Authors:** Milene Peterson, Joe G. L. Hunter, Zoran Gatalica, Inga Rose, Phillip Stafford, Chris W. Diehnelt

**Affiliations:** 1 Robust Diagnostics, LLC, Chandler, AZ, United States; 2 Reference Medicine, Inc., Phoenix, AZ, United States; 3 Arizona State University, Tempe, AZ, United States

**Keywords:** analytical validation, EBV, Epstein–Barr virus, multi-antigen serology, multiplexed immunoassay, peptide microarray, pre-analytical variables, streck blood collection tubes

## Abstract

**Background:**

Epstein–Barr virus (EBV) infects more than 95% of adults and is associated with outcomes ranging from asymptomatic latency to infectious mononucleosis (IM), malignancies, and multiple sclerosis (MS). Conventional ELISA-based EBV serology is often restricted to a small number of soluble, whole-protein antigens and does not preserve information regarding the specific linear regions of antigen recognition that contribute to the overall antibody response. Multiplexed peptide microarrays address this limitation, but their use in clinical practice requires characterization of their analytical performance and behavior across the diverse conditions encountered in practical use.

**Methods:**

Here we report the analytical validation of a 108-peptide microarray spanning nine EBV proteins, applied to 329 specimens collected as serum, EDTA plasma, and Streck cell-free DNA (cfDNA) blood collection tubes.

**Results:**

Within-block and between-slide reproducibility, detection thresholds, and minimum detectable fold-change were consistent with established peptide-array benchmarks, and composite antigen scores correlated with ELISA assays for EBNA-1, VCA-p18, and EA-D. IgG signals were stable across collection matrices, whereas IgM was selectively attenuated in Streck plasma, indicating that fixative-containing tubes should be approached cautiously when IgM is the readout of interest. When applied to IM, MS, and non-MS donors, the peptide array consistently detected broad EBV humoral reactivity. In this proof-of-concept disease-cohort analysis, the array recapitulated biologically relevant EBNA-1 C-terminal reactivity patterns previously associated with MS-related molecular mimicry, but array-wide EBV reactivity did not yield a simple MS-discriminating signature. Although EBV reactivity has been reported to distinguish MS from non-MS cohorts, only a very small set of EBV epitopes was cohort-associated in our data, indicating that platform-level seropositivity reflects EBV exposure rather than an obvious MS-specific signal.

**Conclusion:**

These findings support the platform as an analytically characterized, peptide-level EBV serology tool for translational research.

## 1 Introduction

Epstein–Barr virus (EBV) is a γ-herpesvirus that infects more than 95% of adults worldwide and persists for life as a latent infection of B cells. While most primary infections are clinically silent, EBV is the causative agent of infectious mononucleosis (IM), a recognized cofactor in lymphoid and epithelial malignancies, and has been established as a necessary but not sufficient cause for the development of multiple sclerosis (MS), with seroconversion typically preceding clinical disease by a decade or more. EBV is also associated with additional autoimmune conditions, including systemic lupus erythematosus (1–6).

Conventional EBV serology relies on enzyme-linked immunosorbent assays (ELISA) for a limited panel of whole-protein antigens, typically EBNA-1, viral capsid antigen (VCA), and early antigen-D (EA-D) (6, 7). These assays effectively classify exposure and infection stage but do not resolve antibody responses across the broader EBV proteome. Consequently, questions regarding how EBV-directed humoral responses are distributed across antigens, concentrated within specific regions of individual proteins, or differ between clinical states are difficult to address using conventional formats. Because whole-protein assays aggregate antibody binding across an entire antigen into a single measurement, individuals with similar antigen-level serology may recognize different linear regions of the same protein, information that is not retained in conventional ELISA measurements (8–10).

Multiplexed peptide microarrays provide a complementary approach by enabling simultaneous, semi-quantitative measurement of antibody reactivity against many short linear epitopes (8–14). For such platforms to support clinical research, two requirements must be met: first, analytical performance such as reproducibility, sensitivity, dynamic range, and the sources of technical variance must be characterized; second, the platform must behave predictably across the conditions encountered in clinical workflows. Most blood is collected as serum, EDTA plasma, and increasingly common cell-free DNA (cfDNA) blood collection tubes that incorporate formaldehyde-releasing stabilizers for nucleic acid preservation (15–20). Whether such stabilizers materially affect multiplexed antibody measurement is incompletely characterized and has direct implications for studies that rely on remnant cfDNA-tube specimens.

In this work, we report the analytical validation of an EBV peptide microarray comprising 108 peptides spanning nine major EBV antigens. The novelty of this study lies in the comprehensive analytical characterization of a multi-antigen EBV peptide microarray under conditions relevant to clinical and translational research applications. By evaluating reproducibility, detection characteristics, technical sources of variation, ELISA concordance, and matrix-dependent effects across serum, K2 EDTA plasma, and Streck cfDNA plasma, this work establishes a framework for broader translational use of peptide-array-based EBV serology. Through systematic comparison of these specimen types, we demonstrate reproducible, high-resolution antibody profiling while preserving peptide-level resolution beyond that obtainable with traditional antigen-level assays. We further benchmark composite antigen scores against conventional EBV serology and demonstrate broad EBV humoral reactivity across clinically heterogeneous cohorts including infectious mononucleosis (IM), multiple sclerosis (MS), and non-MS donors. The disease-cohort analyses were included to evaluate whether the analytically characterized platform captures known features of EBV immunobiology and to generate hypotheses for future studies. More detailed disease-specific investigations, including MS-focused multivariable modeling and biomarker-development studies, will be reported separately.

## 2 Materials and methods

### 2.1 Study cohorts and specimen collection

A total of 329 de-identified human blood specimens were used, comprising people with MS (pwMS, n=74), non-MS EBV+ controls (n=194), infectious mononucleosis (IM, n=39), colorectal cancer (CRC, n=12), and non-CRC healthy controls (HC, n=10) (**Table 1**). Specimens were procured from multiple commercial vendors and academic biorepositories. A matrix sub-study evaluated pre-analytical effects across three collection methods: Streck cfDNA blood collection tubes (n=22; formaldehyde-releasing stabilizer), K2 EDTA plasma tubes (n=18), and serum separator tubes (n=10). The Streck sub-cohort comprised CRC patients (n=12) and non-cancer controls (n=10) and was used to assess matrix-associated effects on IgG and IgM detection. For benchmarking against established serology, paired ELISA results for EBNA-1 IgG, VCA-p18 IgG/IgM, and EA-D IgG were obtained from a SeraCare reference cohort and a Discovery Life Sciences validation cohort. Laboratory personnel were not formally blinded to clinical group assignment, which is acknowledged as a limitation; however, array processing and feature extraction were performed using standardized workflows before group-level statistical analysis.

**Table 1:** Characteristics of the study cohort.

Characteristic	Non-MS	MS¹	IM	Non-CRC	CRC
Participants, n	194	74	39	10	12
Female, n	85	34	14	7	9
Male, n	69	9	5	3	3
Not identified, n	40	31	20	0	0
Age (± SD), years	42.2 ± 16.6	45.1 ± 13.2	32.2 ± 17.4	57.5 ± 5.5	61.5 ± 11.8
K2 EDTA, n	50	8	0	0	0
Plasma, n	20	66	0	0	0
Serum, n	124	0	39	0	0
Streck plasma, n	0	0	0	10	12

MS, multiple sclerosis; IM, infectious mononucleosis; CRC, colorectal cancer. ¹For the MS cohort, clinical phenotype and treatment status were not available. Specimen sources included Sonora Quest, SeraCare, Precision for Medicine, Discovery Life Sciences (DLS), Creative Testing Solutions (CTS), Arizona State University, Banner Health, Boca Biolistics, and Reference Medicine.

### 2.2 Peptide array design

The array comprised 108 synthetic peptides representing immunodominant regions from nine EBV proteins: EBNA-1, EBNA-2, EBNA-3, EBNA-4, EBNA-6, BMRF1 (EA-D), BLRF2 (VCA-p23), BFRF3 (VCA-p18), and BLLF1 (gp350/220), covering both latent and lytic phases (**Supplementary Table 1**) (21–23). Peptides were 15-mers with a 10-residue tiling overlap and a C-terminal Gly-Lys-Cys linker for oriented surface attachment (8). Peptides were synthesized by MilliporeSigma (The Woodlands, TX) and spotted in triplicate on Schott Nexterion H functionalized slides (Schott Applied Microarrays, Tempe, AZ). Positive control features included immunodominant peptides from polio and other common vaccine-derived antigens to serve as internal benchmarks of assay performance.

### 2.3 Binding assay and image acquisition

Specimens were diluted 1:400 in incubation buffer and incubated with array slides overnight at 4 °C with continuous agitation. The 1:400 working dilution was selected from a five-point dilution series to maximize signal-to-threshold ratio while avoiding high-dose hook effects observed in EBV-reactivated donors at 1:200 (**Supplementary Figure 5**). After PBST washes, antibody binding was detected with DyLight555-conjugated goat anti-human IgG (1:1, 000) and DyLight650-conjugated goat anti-human IgM (1:10, 000), incubated 1 h at room temperature in the dark. Slides were washed sequentially with PBST, PBS, and HPLC-grade water, dried under nitrogen, and scanned on an Agilent DNA Microarray Scanner at 550 nm (IgG) and 640 nm (IgM). Fluorescence intensities were extracted with Spotxel (SICASYS Software, Germersheim, Germany); per-feature mean, median, standard deviation, and CV were retained for downstream analysis.

### 2.4 Data processing and statistical analysis

Analyses were performed in JMP (18.0, SAS Institute, Cary, NC) and Microsoft Excel (Microsoft, Redmond, WA). Analytical performance was assessed using two QC datasets: 54 technical replicate slides of a single control specimen (RBD-22-3014) spanning eight experiments and two laboratory locations (downtown Phoenix and the SanTan facility in Chandler, AZ), and a five-point dilution series (1:200–1:3, 200) of six donors representing EBV-acute, EBV-latent, EBV-reactivated, and EBV-seronegative states. Within-block CV, between-slide CV, detection threshold (mean + 2 SD of slide-global background), limit of detection (probit regression on the dilution series at P[detect]=0.95), and minimum detectable fold-change (MDFC; estimated two ways, an empirical, distribution-free 95th percentile of |log_2_ ratio| across replicate-slide pairs, and a parametric power analysis at triplicate N = 3 replicates) were computed. Variance decomposition by linear mixed model on log_2_(MFU) evaluated laboratory location, slide-manufacturing batch, and scanner-calibration contributions, with per-feature Type II F-tests and Benjamini–Hochberg FDR correction (q<0.05). Full analytical methodology, equations, and per-factor results are provided in Supplemental Data. Formal *post hoc* batch-correction algorithms were not applied to the primary analytical dataset. Instead, batch effects were quantified directly through variance decomposition, which identified slide-manufacturing batch as the dominant technical factor. Future studies will incorporate bridge specimens, lot-anchored normalization strategies, and prospective normalization procedures to further reduce batch-associated variability.

Composite antigen scores (CAS) were created by summing peptide-level fluorescence intensities per antigen, following established methods for aggregating peptide microarray signals against single-protein reference assays (8, 11, 12, 21, 23, 24). Because the number of constituent peptides differs across antigens, antigen-normalized scores were also computed as the per-sample fraction of total EBV reactivity, with antigen-specific maximum composites defined as (number of peptides) × (3 replicates) × (antigen-specific 95th-percentile RFU). Group comparisons used non-parametric tests: Mann–Whitney U for two-group comparisons and Kruskal–Wallis with Dunn’s multiple comparisons for multi-cohort analyses, with Benjamini–Hochberg FDR correction. P-values are reported with 95% confidence intervals where applicable.

For peptide-level analyses, statistical comparisons were performed between study cohorts (IM, MS, and non-MS) with multiple-testing corrections applied. Heatmaps were generated using log_2_-transformed, background-adjusted peptide-isotype features and CAS values after feature-wise scaling. Rows and columns were ordered by marginal sums rather than hierarchical clustering. Under a multiplicative signal structure, marginal-sum ordering recovers the underlying order of row and column values from MFI measurements while preserving interpretable display axes. Samples were therefore displayed according to total EBV reactivity and features according to cumulative reactivity across the study population. This deterministic ordering requires no distance metric or linkage criterion and was selected to facilitate visualization of the range of antibody reactivity across subjects and features. Because marginal-sum ordering is a one-dimensional ranking approach, no disease linkage, cluster assignment, or unsupervised clustering inference is implied by the heatmap display.

For ELISA benchmarking analyses, positivity thresholds for peptide-array features and composite antigen scores (CAS) were established using the SeraCare reference cohort (n=21) and subsequently applied without modification to all available specimens with paired ELISA results. Agreement between ELISA and peptide-array classifications was summarized using true-positive (TP), false-positive (FP), false-negative (FN), and true-negative (TN) counts.

## 3 Results

### 3.1 Array design and analytical performance

**Figure 1:**
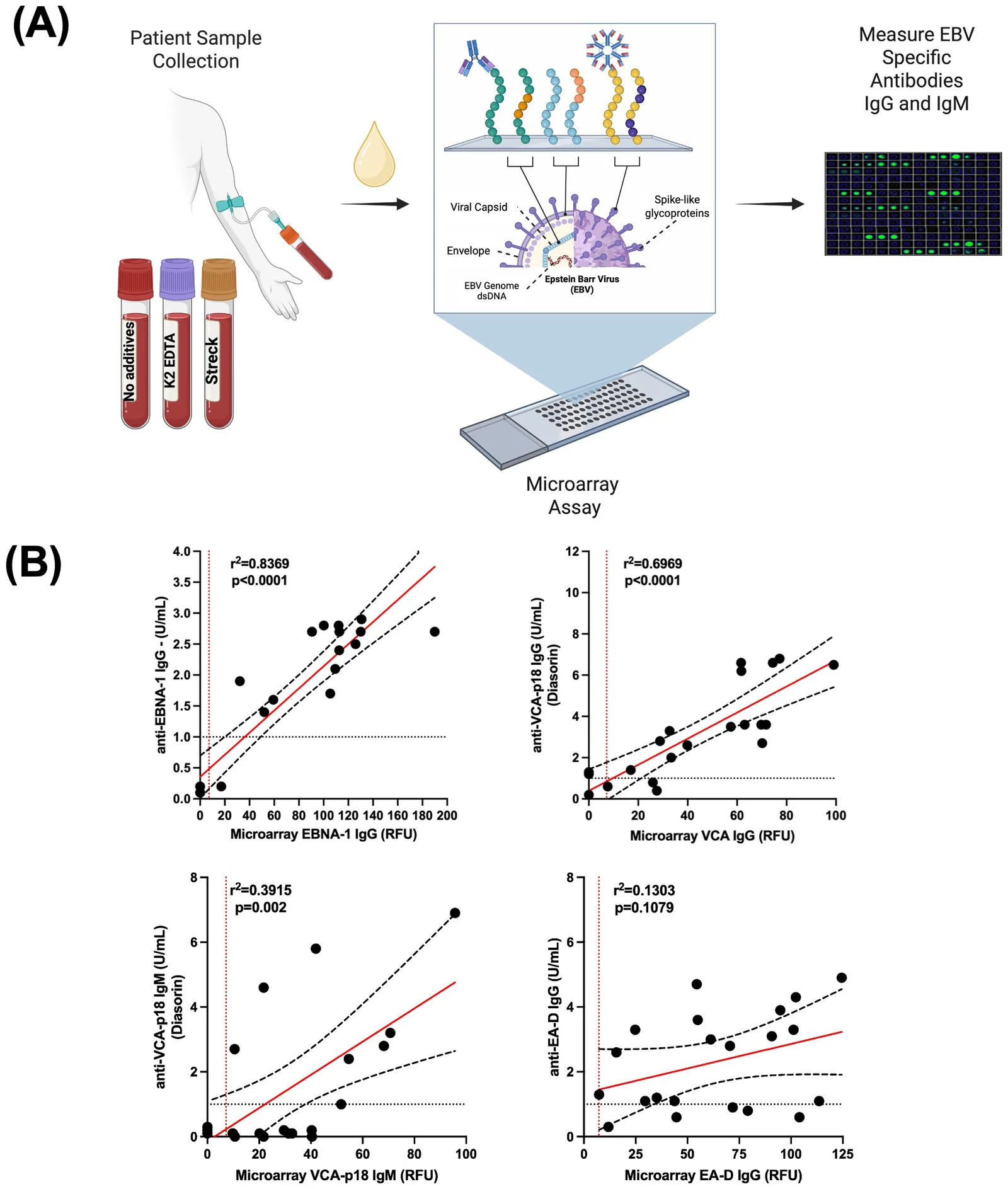
Array design and ELISA concordance. **(A)** Schematic representation of the EBV peptide microarray, comprising 108 overlapping 15-mer peptides spanning nine EBV proteins covering latent and lytic phases. **(B)** Correlation of EBNA-1 IgG, EA-D IgG, and VCA-p18 IgG/IgM composite antigen scores against ELISA in mixed-titer reference samples (LGC SeraCare). Figure created with BioRender.com.

To enable multi-antigen EBV serological profiling, we constructed a peptide microarray of 108 overlapping 15-mer peptides spanning nine EBV proteins across latent and lytic phases (**Figure 1A**; **Supplementary Table 1**). The platform supports simultaneous semi-quantitative measurement of IgG and IgM reactivity at peptide-level resolution within a single assay.

Analytical performance was characterized across 54 technical replicate slides of a single control specimen run at two laboratory locations (downtown Phoenix and the SanTan facility in Chandler, AZ) over eight experimental batches. Within-block reproducibility (median CV ≈14.1% across three spot replicates, and ≈11.1% restricted to above-threshold features) and between-slide reproducibility (median CV ≈11.6%) were consistent with manufacturer specifications for the Nexterion H hydrogel substrate (25). The minimum detectable fold-change was approximately 2-fold and robust across estimation methods: a distribution-free empirical estimate (95^th^ percentile of |log_2_ ratio| across replicate-slide pairs) and a conventional parametric power analysis at N = 3 replicates converged on a similar ≈2-fold value, with the two estimates expected to overlap increasingly as replicate positive control data accumulate. A five-point dilution series defined a working dilution of 1:400, which preserved signal-to-threshold ratio across donor classes while avoiding high-dose hook effects observed in EBV-reactivated donors at 1:200 (**Supplementary Figure 5**). Variance decomposition identified slide-manufacturing batch as the dominant identifiable nuisance factor; laboratory location and scanner calibration contributed minimally. Per-feature Type II F-tests confirmed no significant laboratory-location effect, while batch significantly affected a substantial fraction of detected features at q<0.05, identifying batch control as the primary focus for ongoing process refinement. Full analytical performance details, including LOD, MDFC, spatial-effect analyses, and variance decomposition, are provided in **Supplementary Table 3** and **Supplementary Figures 2**–**S8**.

### 3.2 Concordance with ELISA-based serology

To benchmark the assay against established serology, peptide-array classifications were compared with ELISA calls using positivity thresholds established from the SeraCare reference cohort and subsequently applied to all available specimens with paired ELISA data (**Table 2**). Agreement was evaluated for both individual immunodominant peptides and composite antigen scores (CAS). For EBNA-1 IgG, peptide EBNA1_426–440 demonstrated complete agreement with ELISA classifications, correctly identifying all positive and negative specimens. The corresponding EBNA-1 CAS achieved identical performance, indicating that EBNA-1 CAS classification was largely driven by this dominant peptide. CAS values for EBNA-1 IgG showed strong concordance with ELISA (r² ≈ 0.84, p < 0.0001), in close agreement with prior EBV peptide-to-protein correlations (18). Individual EBNA-1 peptides including EBNA1_426–440 correlated with ELISA at the peptide level (**Supplementary Table 2**; **Supplementary Figure 1**). These results agree with other reports of single EBNA1 peptide correlation with ELISA (26).

**Table 2:** Comparison between ELISA classification and peptide-array classification.

Marker	Method	TP	FP	FN	TN
EBNA1 IgG	EBNA1_426-440-peptide (cutoff >8)	20	0	0	7
	EBNA1 CAS (cutoff >17)	20	0	0	7
EA-D IgG	BMRF1_206-220-peptide (cutoff >7)	15	4	5	3
	EA-D CAS (cutoff >8)	12	5	8	2
VCA IgG	BFRF3_161-175-peptide (cutoff >8)	23	8	4	11
	VCA CAS (cutoff >20)	23	13	4	6
VCA IgM	BFRF3_156-170-IgM peptide (cutoff >8)	13	3	3	15
	VCA-IgM CAS (cutoff >56)	14	7	2	11

ELISA positivity/negativity was assigned using manufacturer-provided interpretive criteria. Positivity thresholds for peptide-array features and composite antigen scores (CAS) were established using the SeraCare reference panel (n=21) and subsequently applied without modification to all available specimens with corresponding ELISA data. Results are summarized as true positives (TP), false positives (FP), false negatives (FN), and true negatives (TN).

Agreement with ELISA was more variable for EA-D and VCA antigens (**Figure 1B**; **Table 2**). For EA-D IgG, BMRF1_206–220 showed higher overall agreement with ELISA than the corresponding CAS, while both approaches displayed lower agreement than observed for EBNA-1. Similarly, for VCA-p18 IgG and IgM, the best-performing individual peptides achieved agreement equal to or greater than the corresponding CAS. Across all antigens evaluated, individual immunodominant peptides matched or exceeded the classification performance of the corresponding antigen-level composite scores.

The weaker correlations observed for VCA-p18 IgM and EA-D IgG likely reflect both biological and assay-format differences. IgM responses are transient and more susceptible to pre-analytical variability, whereas EA-D responses may be temporally heterogeneous and include conformational or discontinuous epitopes that are not fully represented by short linear peptides (21). Cross-vendor comparison of VCA-p18 ELISA (Diasorin and Trinity Biotech) against the array yielded comparable correlations, supporting platform robustness across ELISA reagent sources (**Supplementary Figure 1**). The multiplexed format additionally resolved closely related VCA-p18 and VCA-p23 within a single assay without cross-interference (**Supplementary Figure 1**). Together, these comparisons demonstrate analytical concordance with established serology while providing antigen- and peptide-level resolution beyond what conventional ELISA captures. Accordingly, peptide-level results should be interpreted as complementary to, rather than interchangeable with, whole-protein ELISA. Benchmarking was focused on EBNA-1, VCA, and EA-D because these antigens form the basis of routine clinical EBV serology. Several additional antigens represented on the peptide array do not have broadly adopted clinical ELISA comparators and were included to expand epitope-level characterization of EBV humoral responses beyond the conventional diagnostic panel.

### 3.3 Cross-matrix performance: serum, EDTA plasma, and streck cfDNA tubes

**Figure 2:**
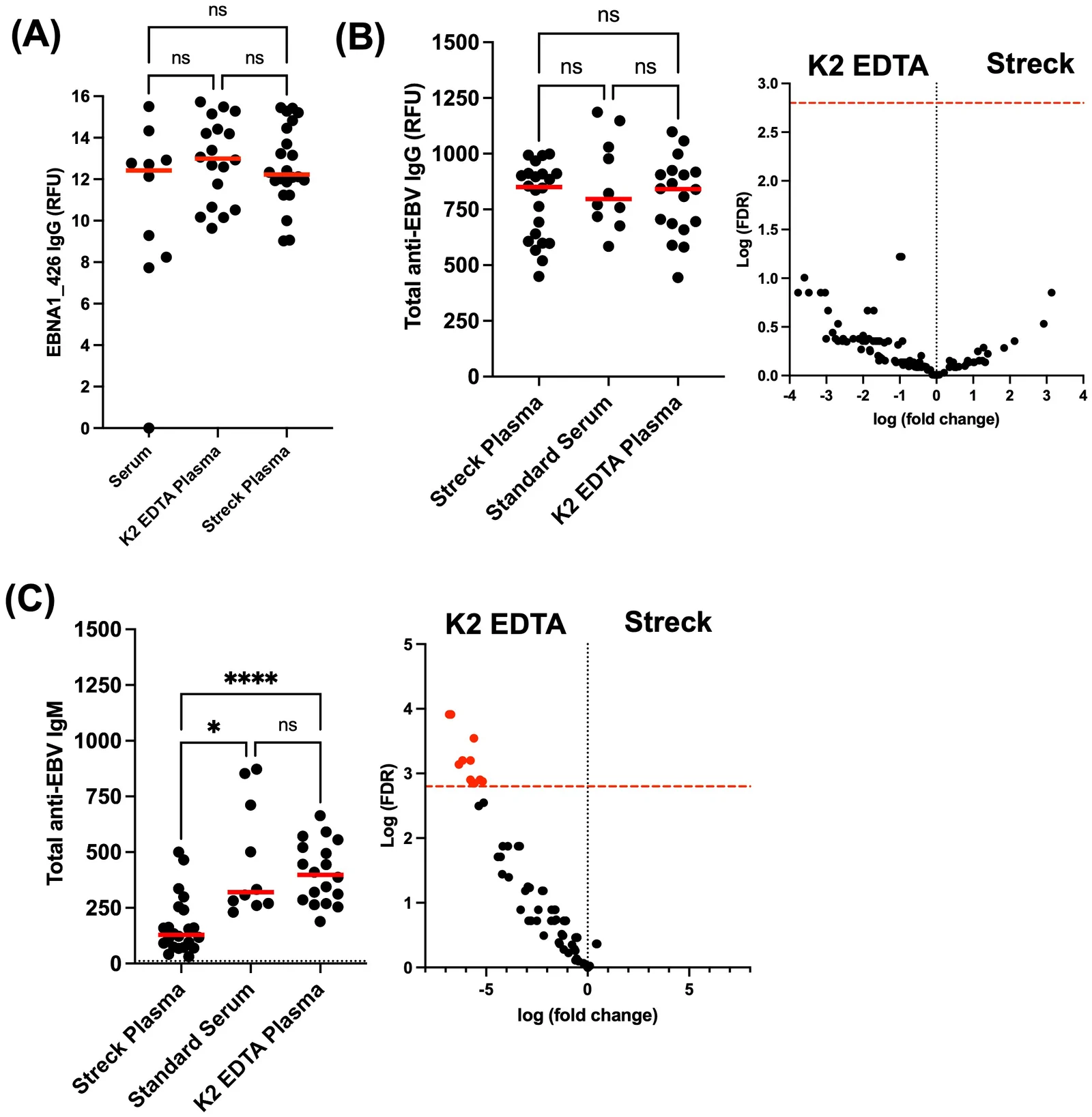
Differential stability of EBV IgG and IgM in Streck cfDNA plasma. Antibody reactivity measured across serum, K2 EDTA plasma, and Streck cfDNA plasma. **(A, B)** Total and antigen-level EBV IgG responses showed no significant differences across matrices, with volcano analysis identifying no significant peptide-level effects. **(C)** Total and antigen-level EBV IgM responses were significantly reduced in Streck plasma, with multiple peptides showing significant attenuation by volcano analysis. P-values calculated using unpaired t-test with Welch’s correction; *p<0.05, ****p<0.0001.

Clinical and translational workflows increasingly include cfDNA blood collection tubes that incorporate formaldehyde-releasing stabilizers. Because remnant samples from these tubes are an attractive source for opportunistic serological analyses, we evaluated whether array measurements remain stable across collection matrices. Across serum, K2 EDTA plasma, and Streck-stabilized plasma, total IgG reactivity to EBV antigens was comparable both at antigen-level and peptide-level (**Figures 2A, B**). IgG signals remained well above the lower limit of quantitation across all three matrices, and volcano plots identified no EBV peptides with significant Streck-associated IgG differences.

IgM signals followed a different pattern. Total IgM reactivity was significantly lower in Streck plasma than in either serum or EDTA plasma, and volcano plot analysis identified multiple EBV peptides with significantly reduced IgM signal in the Streck matrix (**Figure 2C**). IgM signals remained detectable above the IgM lower limit of quantitation (≈11.4 RFU) but were systematically attenuated relative to fresh collection. This is consistent with the known susceptibility of pentameric IgM to fixative-induced cross-linking and conformational change (16, 18). The practical implication for users with remnant Streck-collected material is straightforward: IgG-based EBV profiling on Streck plasma is well supported, whereas IgM measurements from Streck should be interpreted with caution or omitted depending on the study question.

### 3.4 The array captures broad EBV humoral reactivity across clinically heterogeneous cohorts

**Figure 3:**
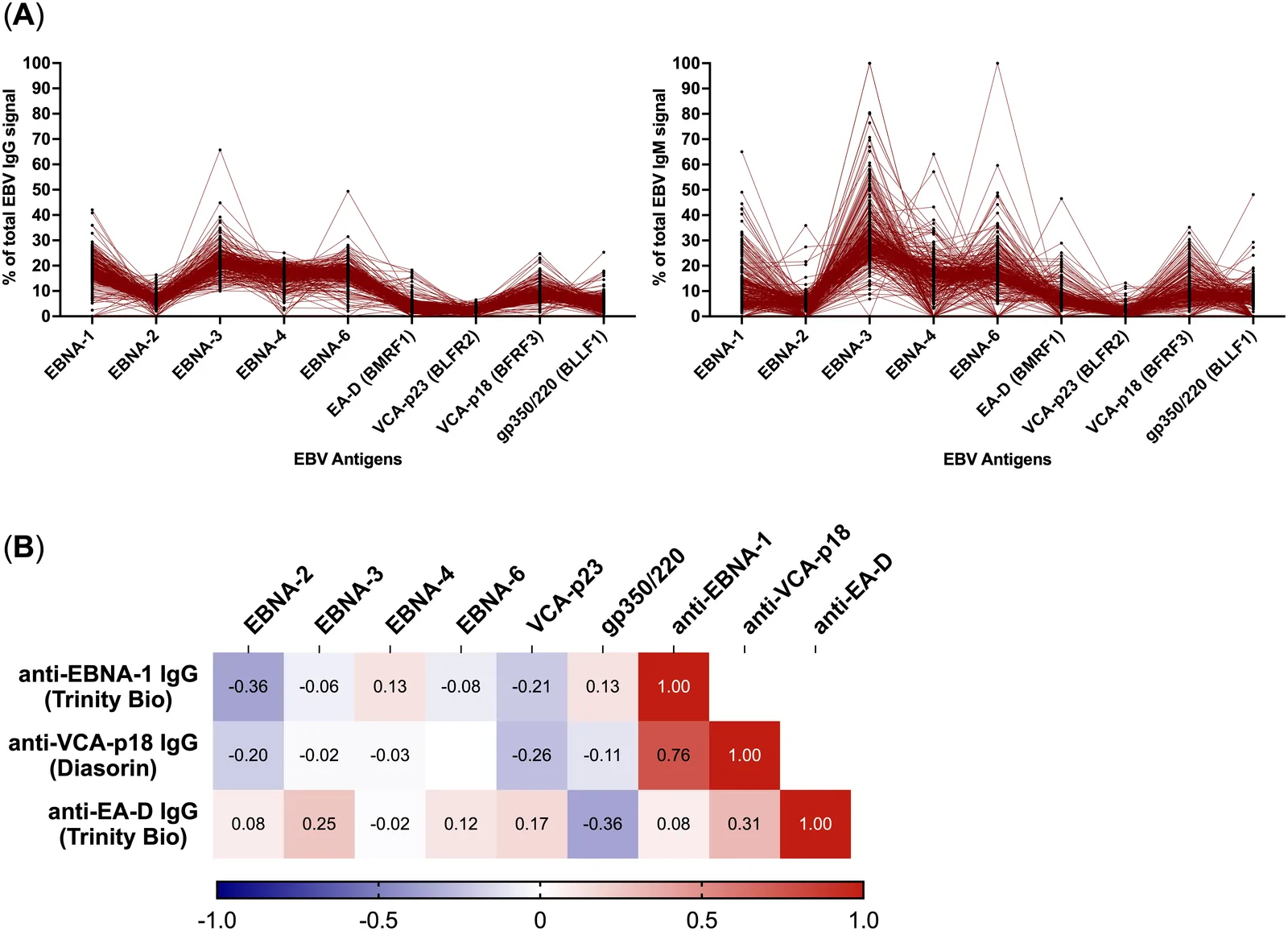
Multi-antigen EBV reactivity profiles across the study cohort. **(A)** Individual-level IgG and IgM antigen composition profiles across nine EBV antigens, expressed as percentage of total EBV antigen signal per sample. Each trace represents a single subject. **(B)** Spearman correlation between microarray CAS values and ELISA titers (U/mL).

**Figure 4:**
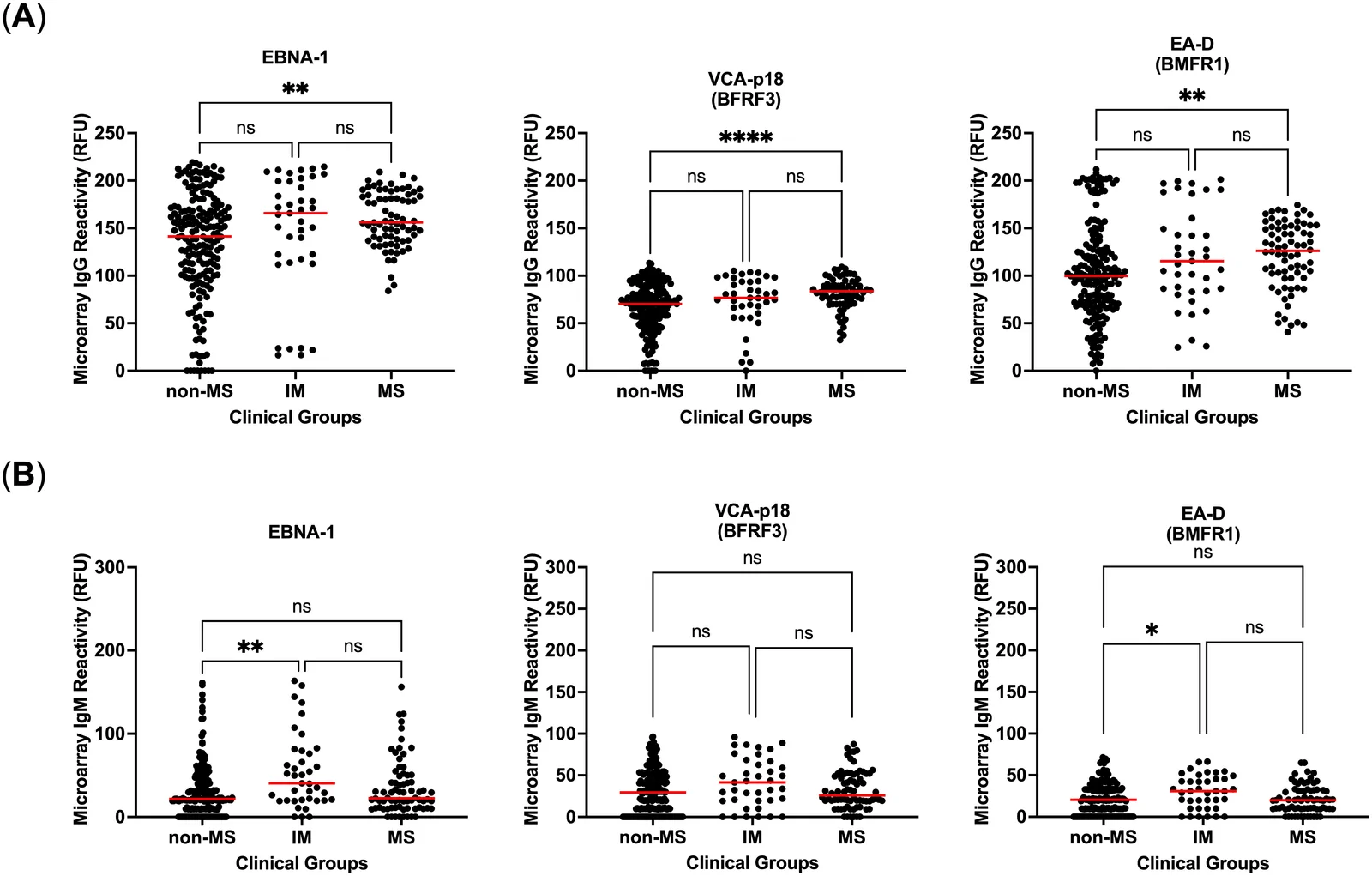
Broad EBV reactivity is detected across IM, MS, and non-MS cohorts. **(A)** IgG reactivity to major EBV antigens (EBNA-1, VCA-p18, EA-D) across non-MS, IM, and MS cohorts. **(B)** IgM reactivity to the same antigens. The Streck sub-cohort was excluded from the IgM analysis based on the matrix effect described in **Figure 2**. Each point represents an individual subject; red bars indicate group medians. Statistical significance assessed by Kruskal–Wallis test with Dunn’s multiple comparisons; ns, not significant; *p<0.05; **p<0.005; ****p<0.0001.

To verify performance across clinical states, we compared antigen-level reactivity profiles in IM, MS, and non-MS donors. All three cohorts showed extensive IgG reactivity across latent and lytic antigens, with the expected hierarchical pattern of dominant EBNA, VCA-p18, and EA-D responses characteristic of EBV-experienced donors (**Figures 3A, B**, **4A**; **Supplementary Figure 9**). IgM responses were lower in magnitude and more variable across individuals, consistent with the expected dominance of class-switched IgG in established EBV infection (**Figure 4B**). Across cohorts, the array consistently captured broad polyclonal EBV reactivity, confirming that the platform is not biased toward any single clinical state.

**Figure 5:**
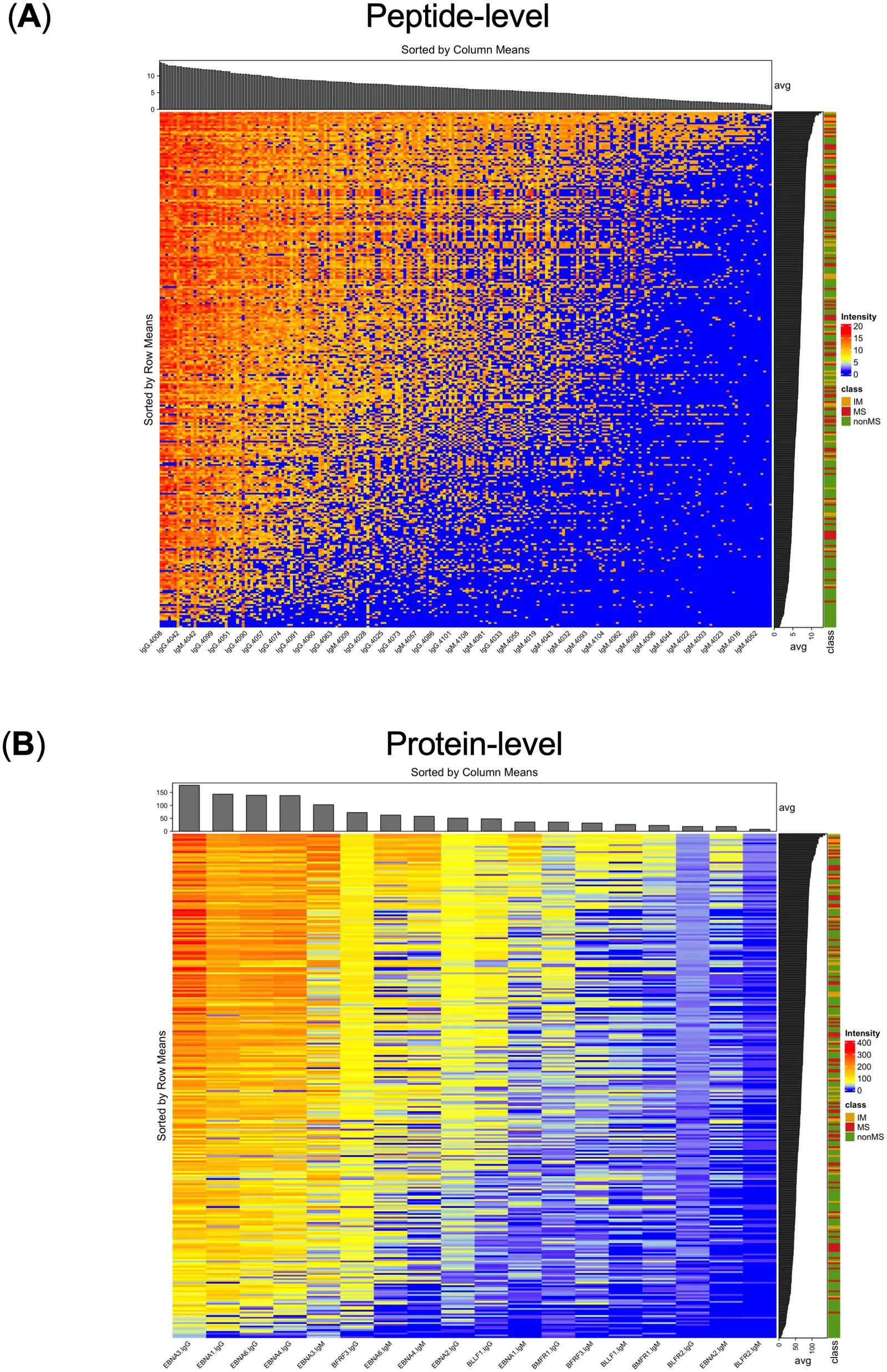
Broad EBV reactivity does not spontaneously cluster by clinical group. Heatmaps of EBV reactivity after log_2_ transformation, background adjustment, and feature-wise scaling. Rows represent individual samples; columns represent **(A)** all 216 peptide-isotype features or **(B)** antigen-level composite scores. Rows and columns were ordered by marginal sums rather than hierarchical clustering. Samples are displayed according to total EBV reactivity and features according to cumulative reactivity across the cohort. No disease linkage or cluster assignment is implied by the ordering. No clear cohort-level separation was evident from array-wide aggregate reactivity.

Although individual antigen-level differences between MS and non-MS donors were detectable, broad EBV-wide reactivity did not, by itself, provide a discriminator of MS from non-MS donors. Visualization of peptide- and antigen-level reactivity after marginal-sum ordering revealed substantial overlap between MS, IM, and non-MS samples, with no clear disease-associated separation evident across the dominant axis of EBV reactivity (**Figures 5A, B**). This observation is consistent with the near-universal prevalence of EBV exposure in adults: the breadth of array-wide EBV reactivity reflects exposure history rather than MS-specific signal, and disease-associated structure, if present, requires analytical approaches beyond aggregate antigen-level summaries.

**Figure 6:**
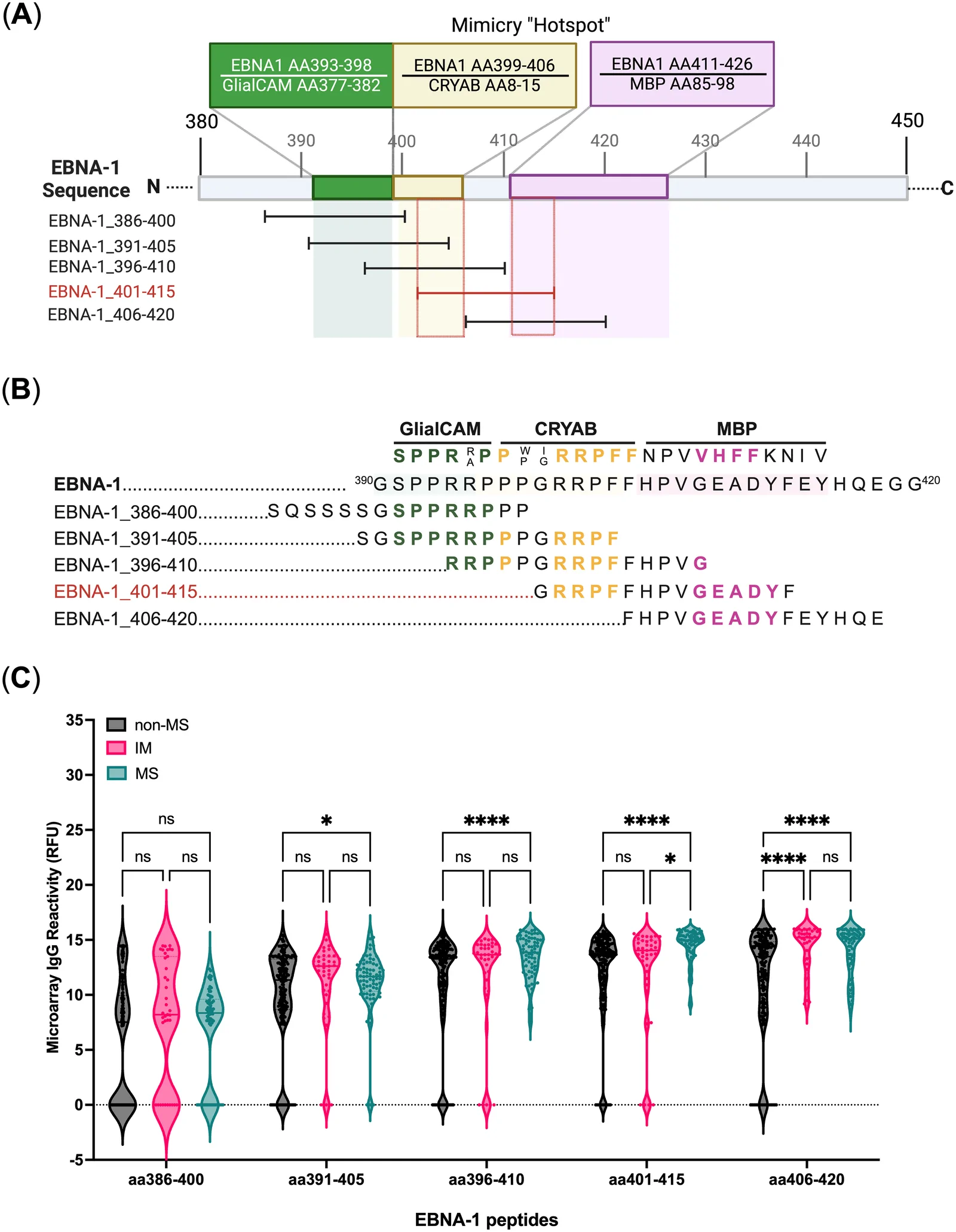
The array recapitulates the previously reported EBNA-1 C-terminal pattern. **(A)** Sequence alignment of EBNA-1 with published host mimicry domains in GlialCAM, alpha-B crystallin (CRYAB), and myelin basic protein (MBP); tiled microarray peptides spanning the C-terminal region are indicated. **(B)** Detailed alignment of EBNA-1 (aa 390–420) with array peptides and published cross-reactive motifs for GlialCAM_393–398, CRYAB_400–410, and MBP_411–419. **(C)** IgG reactivity (RFU) for EBNA-1 peptides across clinical cohorts. Statistical significance by mixed-effects analysis with multiple comparisons; ns, non-significant; *p<0.05; ****p<0.0001.

As a positive control for the array’s ability to capture previously reported EBV serological patterns, we examined the EBNA-1 C-terminal region (residues 386–420), which contains sequences that were previously associated with MS (4, 18, 23–25). These sequences overlap regions implicated in molecular mimicry and cross-reactive antibody responses involving GlialCAM, CRYAB, and MBP. Tiled peptides spanning this region (EBNA1_396-410, EBNA1_401-415, and EBNA1_406-420) showed cohort-associated differences that were generally consistent with prior reports (**Figures 6A–C**), indicating that the array reproduces established EBNA-1 reactivity patterns. These observations should not be interpreted as evidence of a single, simple MS-discriminating serological signature; rather, they suggest that the platform can detect previously reported cohort-level differences within the context of a broader EBV-reactive background. A more detailed analysis of MS-associated serological patterns across additional MS cohorts, including multivariable modeling, will be reported separately.

## 4 Discussion

We report the analytical validation of a 108-peptide microarray spanning nine EBV antigens. The platform supports multi-antigen, epitope-level serological profiling with reproducibility, detection performance, and ELISA concordance consistent with established benchmarks. Within-block and between-slide CVs were within the expected range, and the approximately 2-fold minimum detectable fold-change was concordant across empirical and parametric (N = 3) estimates. Together with ELISA concordance across EBNA-1, VCA-p18, and EA-D, these results position the platform as suitable for downstream EBV research applications. Slide-manufacturing batch was the dominant identifiable technical factor and is the appropriate focus for ongoing process control; laboratory location and scanner calibration contributed minimally under the conditions tested.

The analytical performance observed in this study supports use of the platform for exploratory and translational serology applications, particularly those aimed at detecting moderate-to-large differences in antibody reactivity. Additional validation and standardization will be required before consideration for regulated or diagnostic use. An important implication of the validation is that the peptide array provides information that is complementary to, rather than a replacement for conventional ELISA. Whole-protein ELISAs are well established for assessing EBV exposure and infection stage, whereas the peptide array provides higher-resolution characterization of antibody reactivity across individual linear epitopes within and across antigens. This peptide-level profiling can identify differences in the specific linear regions recognized by antibodies that may not be apparent from whole-protein measurements alone. As a result, individuals with similar ELISA measurements may exhibit distinct peptide-level reactivity patterns despite comparable antigen-level serology. The multiplexed format preserves information regarding the specific linear regions recognized by an individual’s antibody response, information that is not captured by conventional whole-protein ELISAs. Individuals with similar antigen-level serology may exhibit distinct peptide-level reactivity patterns, enabling higher-resolution characterization of EBV humoral responses, longitudinal monitoring of antibody responses, comparative analysis of latent and lytic antigen targeting, and hypothesis generation in EBV-associated diseases.

The linear-peptide format also represents an important limitation of the platform. Because the assay measures antibody binding to short, surface-immobilized linear peptides, it does not fully represent epitopes that depend on native protein folding, glycosylation, quaternary structure, or discontinuous conformational features. This limitation may contribute to differences observed between peptide-array and whole-protein serological measurements for some antigens. Accordingly, peptide-array results should be interpreted as a higher-resolution assessment of the linear-epitope component of EBV humoral immunity rather than as a direct substitute for conventional protein-based serology.

Cross-matrix evaluation provides practical guidance for studies that incorporate mixed or remnant clinical specimens. IgG reactivity was indistinguishable across serum, EDTA plasma, and Streck cfDNA tubes, supporting use of the array on opportunistic samples drawn into liquid-biopsy collection systems. IgM was selectively attenuated in Streck plasma but remained detectable above the IgM lower limit of quantitation. The pattern is mechanistically consistent with the known susceptibility of pentameric IgM to fixative-induced cross-linking and is in line with broader pre-analytical considerations for IgM measurement (16, 18). For investigators relying on remnant Streck-tube material, the practical recommendation is direct: IgG measurements are robust, while IgM should be interpreted with caution or omitted depending on the analytical question. This distinction is important because it allows existing biorepositories of Streck-tube samples, often used for cfDNA NGS testing, to be productively repurposed for EBV IgG serology while transparently flagging the IgM limitation.

Application of the platform to clinically heterogeneous cohorts confirmed that broad EBV humoral reactivity is detected across IM, MS, and non-MS donors. The array readily captures the dominant latent and lytic antibody responses that define EBV exposure, and it does so consistently across cohorts. The data show that array-wide EBV reactivity does not separate MS from non-MS donors by itself. Visualization of peptide- and antigen-level reactivity after marginal-sum ordering demonstrated substantial overlap in reactivity across MS, IM, and non-MS samples, with no clear disease-associated separation along the dominant axis of EBV reactivity. This observation is unsurprising given the near-universal prevalence of EBV exposure in adults, but it has direct implications for biomarker development: a high-resolution multiplexed measurement is necessary but not sufficient for extraction of MS-relevant signals, and platform-level “EBV positivity” should not be conflated with an MS-specific serological signature. The EBNA-1 C-terminal tiling data are consistent with this interpretation. Our observations recapitulate previously reported cohort-level patterns in the C-terminal region (23, 27–29), but also show that these signals are embedded within a broadly EBV-reactive background and are not readily captured by aggregate antigen-level measurements alone.

The disease-cohort findings should be interpreted within the context of the study design. The IM, MS, and non-MS analyses were included as exploratory biological applications of the analytically validated platform rather than as a dedicated diagnostic investigation. Several factors may have influenced the ability to detect cohort-associated peptide patterns, including cohort size, heterogeneous specimen sources and incomplete clinical annotation. In addition, specimen matrix, age distribution, and demographic metadata were not fully balanced across cohorts. Within these limitations, only a small number of peptides showed differences between cohorts, suggesting that broad EBV seroreactivity and unsupervised clustering alone are insufficient to define a distinct MS-associated serological signature in this dataset.

The analytical validation presented here establishes reproducibility, detection characteristics, matrix effects, and the major sources of technical variation for this EBV peptide microarray. Additional studies will be needed to support broader translational applications, including implementation of standardized reference materials, lot-to-lot comparability procedures, and assessment of reproducibility across operators and laboratories. Because slide-manufacturing batch represents the largest identifiable technical factor, future studies will incorporate bridge specimens, batch-aware study designs, and normalization approaches anchored to common reference samples and control features. Such efforts will facilitate evaluation of the platform in larger multi-site studies and biomarker-development settings.

Taken together, the platform performs as a validated multi-antigen, multi-isotype EBV serology tool. Its analytical characteristics, ELISA concordance, and demonstrated compatibility with multiple specimen types including remnant Streck plasma with noted limitation, support its use for EBV research, including longitudinal studies in MS and other EBV-associated diseases.

Although the current data do not support use as a standalone MS diagnostic assay, they demonstrate the potential utility of the platform for characterizing linear-epitope reactivity, identifying candidate immunodominant regions, monitoring response breadth over time, and generating hypotheses for future biomarker investigations. Future studies will further evaluate how peptide-level EBV reactivity relates to clinical, demographic, and host-immunogenetic factors. More detailed disease-focused analyses built upon this analytical foundation are ongoing and will be reported separately.

## Data Availability

The raw data supporting the conclusions of this article will be made available by the authors upon reasonable request to the corresponding author, subject to standard data use agreements. Supplemental tables provide peptide content and summary analytical metrics supporting the findings reported in this article.
