# Supplementary material for: Analytical validation of a multiplexed peptide microarray for multi-antigen Epstein–Barr virus serological profiling across diverse clinical specimens
Source: Front Immunol. 2026 Sep 3;17:1919983. doi: 10.3389/fimmu.2026.1919983 (PMC13582180; doi:10.3389/fimmu.2026.1919983)
Supplement: Supplementary file 1 [file DataSheet1.pdf]

Supplemental Information for: Analytical Validation of a Multiplexed Peptide Microarray for Multi-Antigen Epstein–Barr Virus Serological Profiling Across Diverse Clinical Specimens

Supplemental Figures and Tables
Supplemental Methods
Table S1: Peptides on Microarray
Table S2: EBV Microarray Response Distribution**.**
Table S3: Analytical performance summary.
Figure S1: Cross-platform validation of EBV VCA-p18 using microarray vs. epitope-level ELISA correlations
Figure S2: Median within-block replicate CV across a slide
Figure S3: Per slide intensity by slide manufacturing batch/scanner calibration across 54 slides from QC data
Figure S4: CV vs. Intensity for IgG and IgM channels
Figure S5: Signal-to-threshold ratio vs. dilution by EBV class
Figure S6: Per-block intensity surface and block-median-centered residuals
Figure S7: Edge vs. interior spot intensity
Figure S8: Empirical minimum detectable fold-change
Figure S9: Comparative reactivity to EBV antigens across clinical groups

Supplemental Figures and Tables:

This supplemental section provides supporting information for array content, analytical performance, and selected extended cohort analyses. The material documents assay reproducibility, detection characteristics, and additional antigen- and epitope-level observations that support the main findings without extending the central narrative of the manuscript.

Supplemental Methods:

Supplemental analyses were performed on technical replicate slides, dilution-series, and extended cohort-level antigen comparisons. These datasets were used to summarize assay reproducibility, signal-dependent variability, operating-range behavior, and additional antigen-level patterns not included in the main figures.

Data used:

Titration dataset: six samples were assayed at five serial dilutions (1:200, 1:400, 1:800, 1:1600, 1:3,200), including EBV acute, latent, reactivated, and negative donors. This dataset was used to assess dilution response, detection behavior, and working-range selection.

Technical QC dataset: 54 technical replicate slides were used to assess batch- and calibration-associated signal distributions, signal-dependent CV behavior, and other routine assay performance metrics summarized in the supplemental analytical QC figure.

Table S1: *List of overlapping 15-mer peptides across nine EBV proteins (EBNA1, EBNA2, EBNA3, EBNA4, EBNA6, BMRF1, BLRF2, BFRF3, BLLF1) illustrating the coverage by protein by position.*

Table S1. List of Epstein–Barr virus (EBV) Antigens Represented on the Peptide Microarray.

Peptide ID	Peptide Name	Protein	UniProt ID	AA Region	AA Sequence	Reference
4001	EBNA1_66	EBNA1	P03211	66 - 80	HRDGVRRPQKRPSCI	
4002	EBNA1_71	EBNA1	P03211	71 - 85	RRPQKRPSCIGCKGT	
4003	EBNA1_76	EBNA1	P03211	76 - 90	RPSCIGCKGTHGGTG	
4004	EBNA1_146	EBNA1	P03211	146 - 160	GGAGAGGGAGGAGAG	
4005	EBNA1_386	EBNA1	P03211	386 - 400	SQSSSSGSPPRRPPP	
4006	EBNA1_391	EBNA1	P03211	391 - 405	SGSPPRRPPPGRRPF	
4007	EBNA1_396	EBNA1	P03211	396 - 410	RRPPPGRRPFFHPVG	
4008	EBNA1_401	EBNA1	P03211	401 - 415	GRRPFFHPVGEADYF	
4009	EBNA1_406	EBNA1	P03211	406 - 420	FHPVGEADYFEYHQE	

4010	EBNA1_426	EBNA1	P03211	426 - 440	EPDVPPGAIEQGPAD	
4011	EBNA1_431	EBNA1	P03211	431 - 445	PGAIEQGPADDPGEG	
4012	EBNA1_436	EBNA1	P03211	436 - 450	QGPADDPGEGPSTGP	
4013	EBNA1_526	EBNA1	P03211	526 - 540	LAIPQCRLTPLSRLP	
4014	EBNA1_531	EBNA1	P03211	531 - 545	CRLTPLSRLPFGMAP	
4015	EBNA1_621	EBNA1	P03211	621 - 635	GDDGDDGDEGGDGDE	
4016	EBNA1_626	EBNA1	P03211	626 - 640	DGDEGGDGDEGEEGQ	
4017	EBNA2_211	EBNA2	P12978	211 - 225	LTVPPRPTRPTTLPP	
4018	EBNA2_216	EBNA2	P12978	216 - 230	RPTRPTTLPPTPLLT	(14)
4019	EBNA2_221	EBNA2	P12978	221 - 235	TTLPPTPLLTVLQRP	(14)
4020	EBNA2_466	EBNA2	P12978	466 - 480	ESPSSDEDYVEGPSK	(14)
4021	EBNA2_471	EBNA2	P12978	471 - 485	DEDYVEGPSKRPRPS	(14)
4022	EBNA2_476	EBNA2	P12978	476 - 490	DYVEGPSKRPRPSIQ	(14)
4023	EBNA3_156	EBNA3	P12977	156 - 170	RDQAKWRLQTLAAGW	(14,21)
4024	EBNA3_161	EBNA3	P12977	161 - 175	WRLQTLAAGWPMGYQ	(14,21)
4025	EBNA3_166	EBNA3	P12977	166 - 180	LAAGWPMGYQAYSSW	(14,21)
4026	EBNA3_631	EBNA3	P12977	631 - 645	MEGPLVPEQQMFPGA	(2,21)
4027	EBNA3_636	EBNA3	P12977	636 - 650	VPEQQMFPGAPFSQV	(2,14,21)
4028	EBNA3_641	EBNA3	P12977	641 - 655	MFPGAPFSQVADVVR	(2,14,21)
4029	EBNA3_646	EBNA3	P12977	646 - 660	PFSQVADVVRAPGVP	(2,14,21)
4030	EBNA3_656	EBNA3	P12977	656 - 670	APGVPAMQPQYFDLP	(2,14,21)
4031	EBNA3_661	EBNA3	P12977	661 - 675	AMQPQYFDLPLIQPI	(2,14,21)
4032	EBNA3_666	EBNA3	P12977	666 - 680	YFDLPLIQPISQGAP	(2,14,21)
4033	EBNA3_671	EBNA3	P12977	671 - 685	LIQPISQGAPVAPLR	(2,14,21)
4034	EBNA3_676	EBNA3	P12977	676 - 690	SQGAPVAPLRASMGP	(2,14,21)
4035	EBNA3_721	EBNA3	P12977	721 - 735	PQQPMEGPLVPEQWM	(2,14,21)
4036	EBNA3_726	EBNA3	P12977	726 - 740	EGPLVPEQWMFPGAA	(2,14,21)
4037	EBNA3_731	EBNA3	P12977	731 - 745	PEQWMFPGAALSQSV	(2,14,21)
4038	EBNA3_746	EBNA3	P12977	746 - 760	RPGVAQSQYFDLPLT	(2,14,21)
4039	EBNA3_751	EBNA3	P12977	751 - 765	QSQYFDLPLTQPINH	(2,14,21)
4040	EBNA3_756	EBNA3	P12977	756 - 770	DLPLTQPINHGAPAA	(2,14,21)
4041	EBNA3_761	EBNA3	P12977	761 - 775	QPINHGAPAAHFLHQ	(2,14,21)
4042	EBNA3_826	EBNA3	P12977	826 - 840	VNQYHLSQAAFGLPI	(2,14,21)
4043	EBNA3_831	EBNA3	P12977	831 - 845	LSQAAFGLPIDEDES	(2,14,21)
4044	EBNA3_836	EBNA3	P12977	836 - 850	FGLPIDEDESGEGSD	(2,14,21)
4045	EBNA3_841	EBNA3	P12977	841 - 855	DEDESGEGSDTSEPC	(2,14,21)
4046	EBNA4_16	EBNA4	P03203	16 - 30	GASGSEDPPDYGDQG	(14,21)
4047	EBNA4_21	EBNA4	P03203	21 - 35	EDPPDYGDQGNVTQV	(14,21)
4048	EBNA4_36	EBNA4	P03203	36 - 50	GSEPISPEIGPFELS	(14,21)
4049	EBNA4_41	EBNA4	P03203	41 - 55	SPEIGPFELSAASED	(14,21)
4050	EBNA4_56	EBNA4	P03203	56 - 70	DPQSGPVEENLDAAA	(14,21)
4051	EBNA4_61	EBNA4	P03203	61 - 75	PVEENLDAAAREEEE	(14,21)
4052	EBNA4_306	EBNA4	P03203	306 - 320	RPKAAKPWLRAHPVA	(14,21)
4053	EBNA4_311	EBNA4	P03203	311 - 325	KPWLRAHPVAIPYDD	(14,21)
4054	EBNA4_316	EBNA4	P03203	316 - 330	AHPVAIPYDDPLTNE	(14,21)
4055	EBNA4_386	EBNA4	P03203	386 - 400	QTLPYNPTVYGRPAV	(21)
4056	EBNA4_391	EBNA4	P03203	391 - 405	NPTVYGRPAVFDRKS	(21)
4057	EBNA4_656	EBNA4	P03203	656 - 670	QVEITPYKPTWAQIG	(14,21)
4058	EBNA4_661	EBNA4	P03203	661 - 675	PYKPTWAQIGHIPYQ	(2,14,21)
4059	EBNA4_666	EBNA4	P03203	666 - 680	WAQIGHIPYQPTPTG	(2,14,21)
4060	EBNA4_671	EBNA4	P03203	671 - 685	HIPYQPTPTGPATML	(2,14,21)
4061	EBNA4_706	EBNA4	P03203	706 - 720	PPEVPPVPRQRPRGA	(2,14,21)

4062	EBNA4_711	EBNA4	P03203	711 - 725	PVPRQRPRGAPTPTP	(2,14,21)
4063	EBNA4_716	EBNA4	P03203	716 - 730	RPRGAPTPTPPPQVP	(2,14,21)
4064	EBNA4_721	EBNA4	P03203	721 - 735	PTPTPPPQVPPVPRQ	(2,14,21)
4065	EBNA6_21	EBNA6	P03204	21 - 35	NVQTTGEHDQDPGPG	(2,14,21)
4066	EBNA6_26	EBNA6	P03204	26 - 40	GEHDQDPGPGPPSSG	(2,14,21)
4067	EBNA6_31	EBNA6	P03204	31 - 45	DPGPGPPSSGASERL	(2,14,21)
4068	EBNA6_156	EBNA6	P03204	156 - 170	RRGPLVAEGGVGWRH	(2,21)
4069	EBNA6_161	EBNA6	P03204	161 - 175	VAEGGVGWRHWLLTS	(2,21)
4070	EBNA6_166	EBNA6	P03204	166 - 180	VGWRHWLLTSPSQSW	(2,21)
4071	EBNA6_466	EBNA6	P03204	466 - 480	HPTPVEMPMVILHQP	(2,14,21)
4072	EBNA6_471	EBNA6	P03204	471 - 485	EMPMVILHQPPPVPK	(2,14,21)
4073	EBNA6_556	EBNA6	P03204	556 - 570	PSDTGPPAVGPPAAG	(2,14,21)
4074	EBNA6_561	EBNA6	P03204	561 - 575	PPAVGPPAAGPPAAG	(2,14,21)
4075	EBNA6_566	EBNA6	P03204	566 - 580	PPAAGPPAAGPPAAG	(2,14,21)
4076	EBNA6_706	EBNA6	P03204	706 - 720	SSMSPTQPISHEEQP	(2,21)
4077	EBNA6_711	EBNA6	P03204	711 - 725	TQPISHEEQPRYEDP	(2,21)
4078	EBNA6_716	EBNA6	P03204	716 - 730	HEEQPRYEDPDAPLD	(2,21)
4079	EBNA6_741	EBNA6	P03204	741 - 755	PAPQAPYQGYQEPPA	(2,14,21)
4080	EBNA6_746	EBNA6	P03204	746 - 760	PYQGYQEPPAPQAPY	(2,14,21)
4081	EBNA6_751	EBNA6	P03204	751 - 765	QEPPAPQAPYQGYQE	(2,14,21)
4082	EBNA6_756	EBNA6	P03204	756 - 770	PQAPYQGYQEPPPPQ	(2,14,21)
4083	EBNA6_761	EBNA6	P03204	761 - 775	QGYQEPPPPQAPYQG	(2,14,21)
4084	BMRF1_81	BMRF1	P03191	81 - 95	PAAVSFRNLAYGRTC	(21)
4085	BMRF1_86	BMRF1	P03191	86 - 100	FRNLAYGRTCVLGKE	(21)
4086	BMRF1_106	BMRF1	P03191	106 - 120	VEQASLQFYKRPQGG	(21)
4087	BMRF1_111	BMRF1	P03191	111 - 125	LQFYKRPQGGSRPEF	(21)
4088	BMRF1_206	BMRF1	P03191	206 - 220	CLTLDYKPLSVGPYE	(21)
4089	BMRF1_211	BMRF1	P03191	211 - 225	YKPLSVGPYEAFTGP	(21)
4090	BLRF2_1	BLRF2	P03197	1 - 15	MSAPRKVRLPSVKAV	(14,21)
4091	BLRF2_6	BLRF2	P03197	6 - 20	KVRLPSVKAVDMSME	(14,21)
4092	BFRF3_31	BFRF3	P14348	31 - 45	NQNNLPNDVFREAQR	(21)
4093	BFRF3_36	BFRF3	P14348	36 - 50	PNDVFREAQRSYLVF	(21)
4094	BFRF3_116	BFRF3	P14348	116 - 130	SAPSTAVAQSATPSV	(14,21)
4095	BFRF3_121	BFRF3	P14348	121 - 135	AVAQSATPSVSSSIS	(14,21)
4096	BFRF3_126	BFRF3	P14348	126 - 140	ATPSVSSSISSLRAA	(2,14,21)
4097	BFRF3_131	BFRF3	P14348	131 - 145	SSSISSLRAATSGAT	(2,14,21)
4098	BFRF3_156	BFRF3	P14348	156 - 170	TGSGGGGQPHDTAPR	(2,14,21)
4099	BFRF3_161	BFRF3	P14348	161 - 175	GGQPHDTAPRGARKK	(2,14,21)
4100	BLLF1_21	gp350/220	P03200	21 - 35	EDPGFFNVEIPEFPF	(14,21)
4101	BLLF1_26	gp350/220	P03200	26 - 40	FNVEIPEFPFYPTCN	(14,21)
4102	BLLF1_66	gp350/220	P03200	66 - 80	GQLTPHTKAVYQPRG	(14,21)
4103	BLLF1_71	gp350/220	P03200	71 - 85	HTKAVYQPRGAFGGS	(14,21)
4104	BLLF1_76	gp350/220	P03200	76 - 90	YQPRGAFGGSENATN	(14,21)
4105	BLLF1_731	gp350/220	P03200	731 - 745	SGPGNSSTSTKPGEV	(14,21)
4106	BLLF1_736	gp350/220	P03200	736 - 750	SSTSTKPGEVNVTKG	(14,21)
4107	BLLF1_741	gp350/220	P03200	741 - 755	KPGEVNVTKGTPPQN	(14,21)
4108	BLLF1_746	gp350/220	P03200	746 - 760	NVTKGTPPQNATSPQ	(14,21)

Table S2. EBV Microarray Response Distribution (n=329). *Peptide-level responses are summarized by isotype and protein. Values include median RFU with 95% CI, functional range (5th–95th percentile), fold-change across that range (95th ÷ 5th percentile), replicate coefficient of variation, and maximum antigen composite sum per protein. Where the empirical 5th percentile is zero, the 5th percentile was floored at 1 RFU; therefore, the reported fold-change for those rows equals the 95th-percentile value. All fluorescence values are linear RFU, not* $\log_2$.

Isotype	Protein (# Peptides)	Median [95% CI]	Functional Range (5th - 95th) (RFU)	Fold Change (FC)	CV (%)	Max Antigen Composite
**IgG**	EBNA-1 (16)	149.9 [142.6–154.0]	20.3 – 208.8	10.3x	37	10,022.4
	EBNA-2 (6)	52.2 [48.7–54.0]	8.6 – 76.8	8.9x	41.5	1,382.4
	EBNA-3 (23)	167.5 [159.2–182.7]	48.3 – 298.8	6.2x	44.1	20,617.2
	EBNA-4 (19)	132.8 [125.7–143.0]	27.4 – 243.8	8.9x	49.3	13,896.6
	EBNA-6 (19)	135.6 [127.0–140.9]	32.5 – 233.4	7.2x	44.2	13,303.8
	EA-D (6)	26.3 [24.0–31.0]	5.0 – 83.9	16.8x	71.7	1,510.2
	VCA-p23 (2)	19.25 [18.7–19.9]	0 – 26.3	26.4x	45.3	157.8
	VCA-p18 (8)	73.86 [70.7–76.6]	14.5 – 102.3	7.1x	36.7	2,455.2
	gp350/220 (9)	40.22 [35.0–48.3]	0 – 95.2	95.3x	66.2	856.8
**IgM**	EBNA-1 (16)	21.6 [20.4–28.3]	0 – 115.4	115.4x	103.5	5,539.2
	EBNA-2 (6)	9.8 [9.4–10.5]	0 – 54.6	54.6x	111.1	982.8
	EBNA-3 (23)	78.6 [63.3–95.7]	11.4 – 229	20.0x	72.4	15,801.0
	EBNA-4 (19)	41.2 [30.7–49.8]	0 – 148.9	148.9x	90.5	8,487.3
	EBNA-6 (19)	49.6 [40.6–61.9]	0 – 150.9	150.9x	82.2	8,601.3
	EA-D (6)	20.2 [19.0–20.7]	0 – 54.4	54.4x	83.4	979.2
	VCA-p23 (2)	0 [0–9.3]	0 – 21	21x	120.8	126.0
	VCA-p18 (8)	28.4 [20.9–30.9]	0 – 81.5	81.5x	84.4	1,956.0
	gp350/220 (9)	20.3 [19.3–21.8]	0 – 75.9	75.9x	101.6	2,049.3

Table S3. Analytical performance summary. *Row 1 — Within-block CV: median coefficient of variation across the three spot-replicates printed for each peptide within a sub-array block. Blocks are spotter-defined coordinate regions that allow image-analysis software to locate features. Reflects spot-to-spot reproducibility at the print level.*
Row 2 — Between-slide CV: median and 95% CI of the per-feature CV calculated across all 54 technical replicate slides. Reflects whole-assay reproducibility under routine operating conditions.
Row 3 — Detection threshold: mean + 2 SD of the slide-global background, reported as both mean and median across slides. Defines the cutoff above which a feature is called present.
Row 4 — Limit of detection: per-feature dilution where the probability of detection above the threshold in Row 3 reaches 95%, estimated by probit regression on the dilution series. Median and IQR are reported across features with a probit fit.
Row 5 — Minimum detectable fold-change (MDFC): estimated two complementary ways. The empirical, distribution-free estimate is the 95th percentile of the |log2 ratio| distribution across all replicate-slide pairs, approximating the smallest fold-difference resolvable from technical noise between two single-slide measurements. The parametric estimate is a conventional power analysis at N=3 replicates. Both converge on approximately 2-fold, concordant across empirical and parametric (N=3) estimates.
Row 6 — Total technical variance: combined contribution of all technical sources (residual within-experiment slide-to-slide, between-experiment, peptide-by-experiment interaction, operator, reagent batch, scanner calibration) from a linear mixed model on log2(MFU). Reported as a percentage of total observed variance; the complement reflects between-feature biological signal.
Row 7 – Type II F-test for nuisance factors: 108 peptides are present on the array, comprising 216 total IgG and IgM features. Of these, 187 features were detected above background in the QC data. For each of the 54 samples, lab location, slide batch, and scanner calibration state were recorded as possible confounders. A Type II F-test was computed for each feature using ANOVA for lab location, batch, and calibration in a fitted linear model on log_2(MFU). P-values were corrected using Benjamini-Hochberg FDR per factor at q<0.05. For lab location, 0/187 features passed the significance threshold (effect −0.96 on the log_2(MFU) scale); for batch, 86/187 passed (+1.21 log_2(MFU)); and for scanner calibration, 6/187 passed (+1.04 log_2(MFU)). Slide batch was associated with a significant signal difference for 86 of 187 detected features at q<0.05. The cumulative effect on significance testing is shown in the embedded line graph, where the vertical line represents $p<10^{-04}$. Features to the right of the vertical cutoff did not meet the predefined significance threshold for batch-associated effects.

Metric	Value
Within-block CV (3 spot replicates), median	14.1%
Between-slide CV, detected features, median	11.6±2.5% CI
Calculated detection threshold (mean+2SD background)	80.9 MFU IgG, 170.5 MFU IgM
Mean LoD (probit, P_{detect}=0.95)	1:338 IgG; 1:545 IgM
MDFC: empirical pairwise (p_{95} of |log_2 ratio|) and parametric power at N=3 replicates	≈2-fold (both methods converge)
Variance from all technical sources combined	18.8%
Per-feature nuisance factor F-test: lab location, slide batch, and scanner calibration were recorded per slide. A Type II F-test with BH-FDR q<0.05 was used to determine the percentage of detected features significantly associated with each nuisance factor. The graph shows the test statistic at $p<10^{-04}$, with batch-associated features to the left of the vertical line.	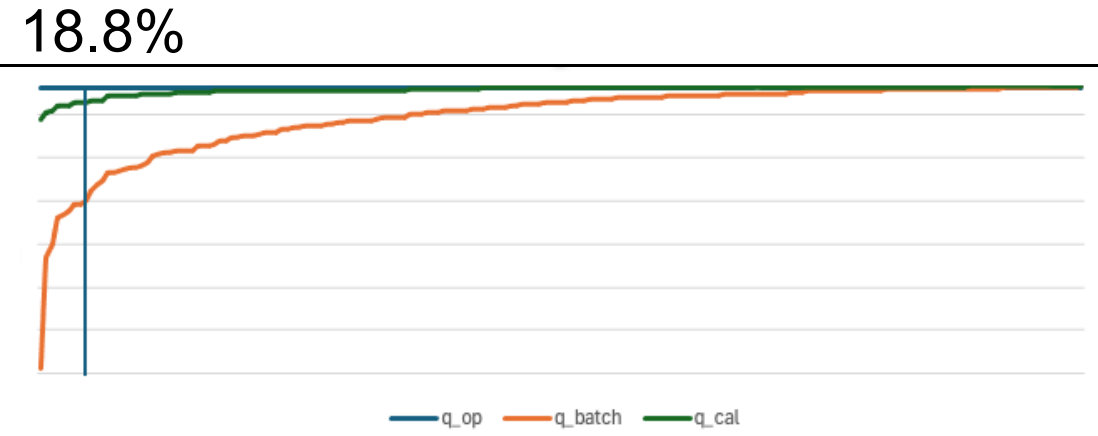 Lab location: 0%, Batch: 46% (86/187), Calibration 3%

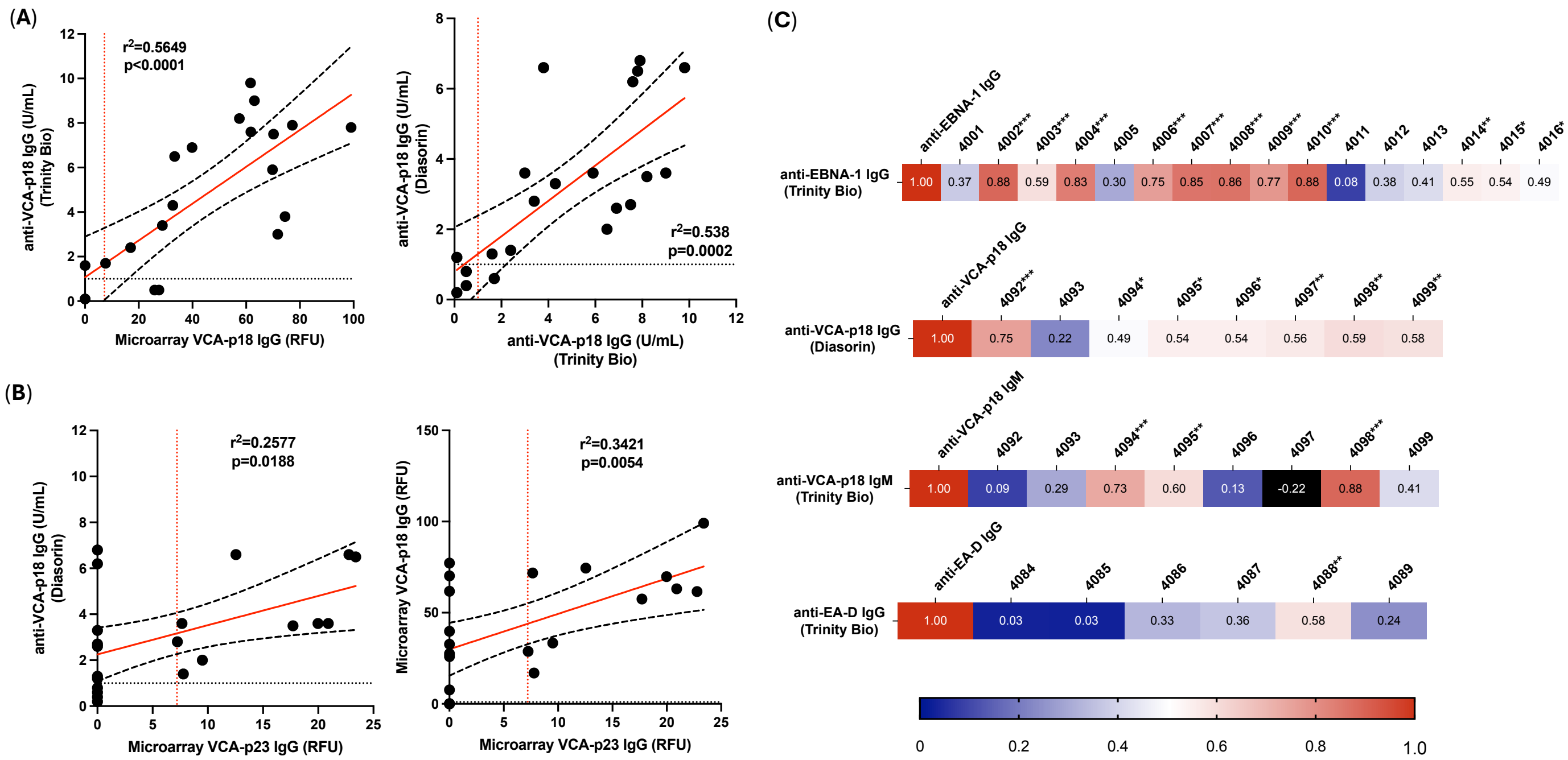

Figure S1. Cross-platform comparison of EBV VCA-p18 using microarray profiling and epitope-level ELISA correlations. ***(A)*** *Cross-vendor comparison of VCA-p18 reactivity shows agreement between array-derived intensities and ELISA data from two commercial providers (Diasorin and Trinity Biotech), supporting cross-platform concordance.* ***(B)*** *Antigen-level comparison between VCA-p18 and VCA-p23 reactivity demonstrates the array's ability to resolve closely related antigens and detect differential antigen-level immune responses.* ***(C)*** *Correlation mapping between epitope-level intensities and broader EBV antigen groups supports the selection of biologically relevant peptides and identifies conserved immunodominant regions within the EBV proteome. Statistical significance for each peptide is indicated within the axis labels based on Spearman correlation analysis. Significance levels are denoted as follows: *, p<0.05; **, p<0.001; ***, p<0.0001. Antigens without asterisks did not reach statistical significance (p≥0.05).*

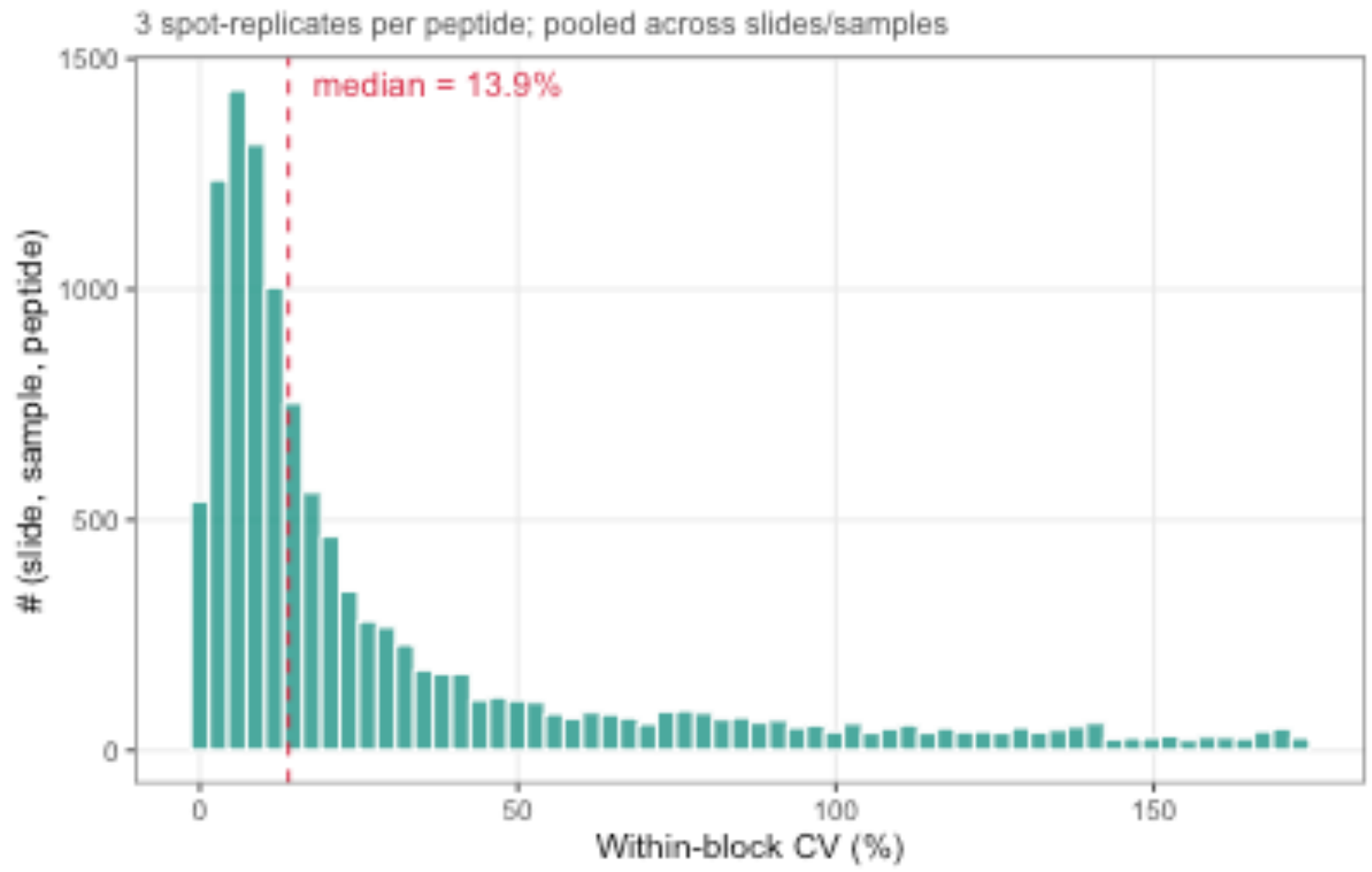

Figure S2. Median within-block replicate CV across a slide. *Coefficient of variation across three spot replicates printed per peptide within a sub-array block, pooled across slides, samples, and peptides, showed a median CV of 14.1% across all features (dashed line). The right tail (>100%) reflects low-signal features where one or more replicates falls near the noise floor. In this range, CV is dominated by background variability rather than true spot-to-spot variation. When restricted to features above the detection threshold, median within-block CV decreased to 11.1%, consistent with manufacturer specifications for the Schott Nexterion H hydrogel substrate (20).*

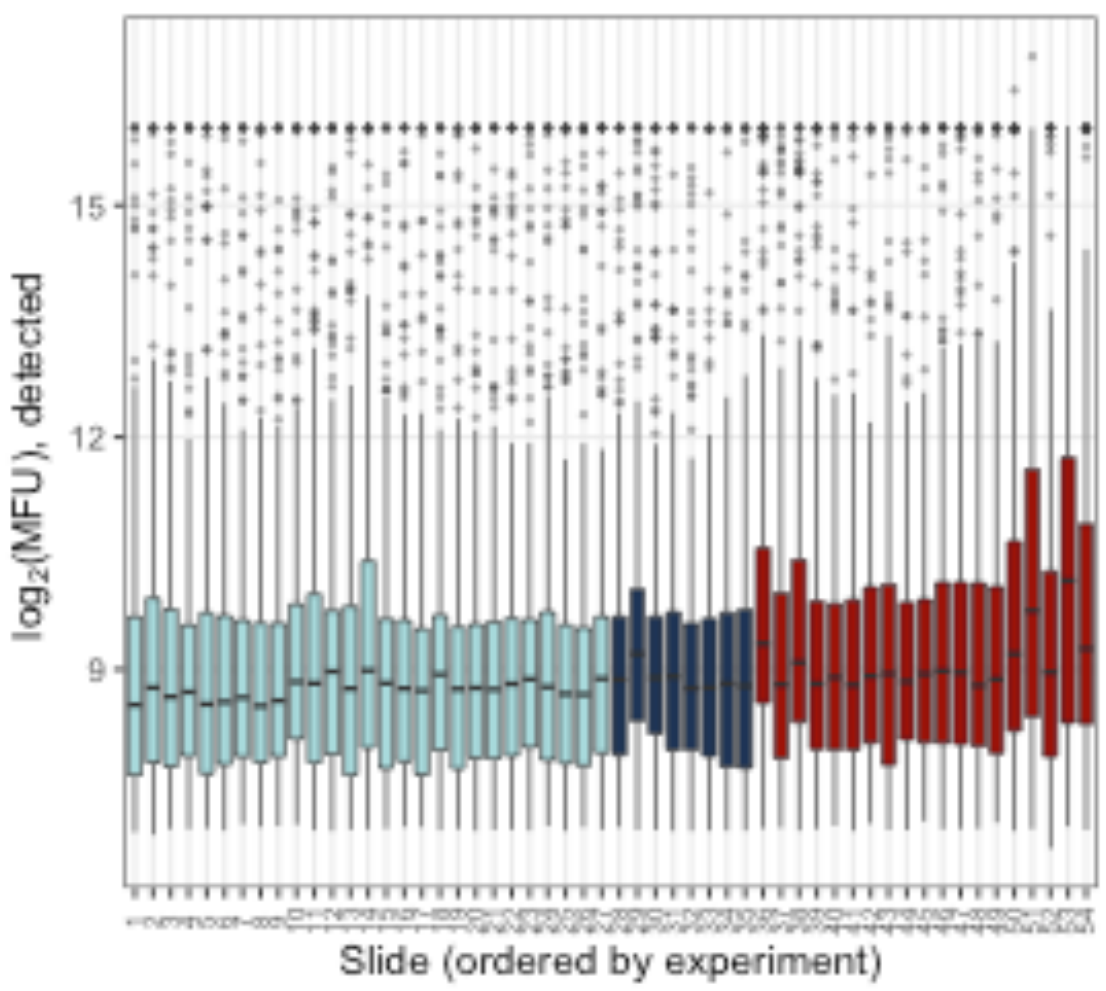

Figure S3. Per-slide intensity by slide manufacturing batch and scanner calibration across 54 QC slides. *Boxplots for MFU (mean fluorescence units) are shown on a log_2 scale for IgG and IgM isotypes combined. Slide batch 1 includes slides colored cyan and dark blue. Scanner calibration 1 includes only slides shown in cyan, whereas scanner calibration 2 includes slides shown in dark blue and red.*

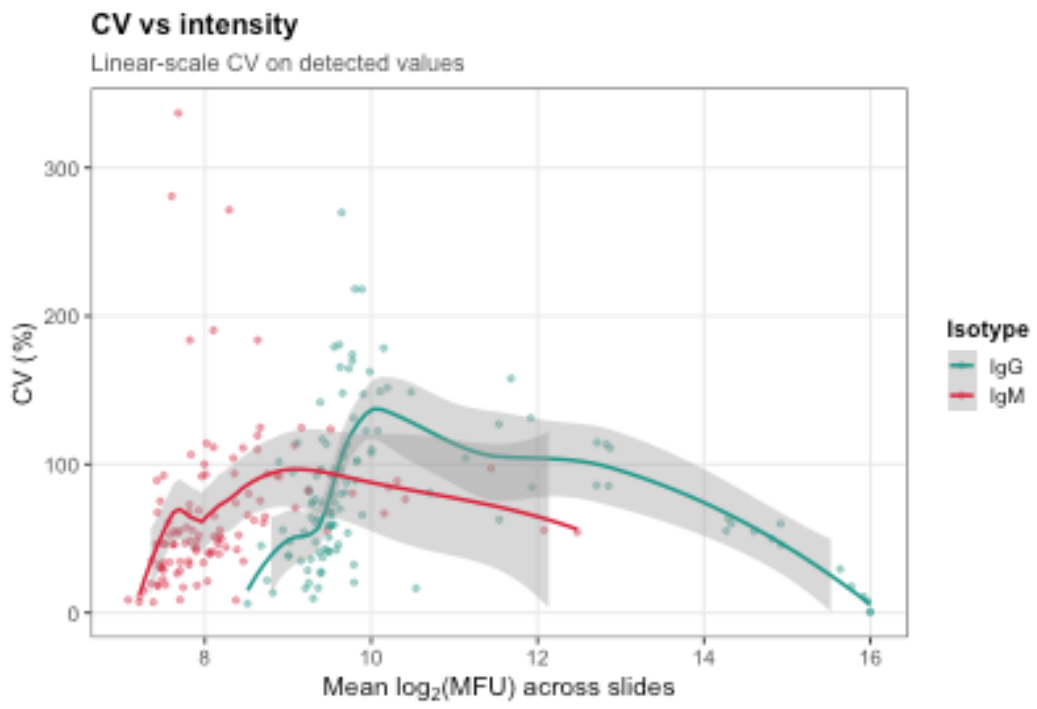

Figure S4. CV vs. intensity for IgG and IgM channels. *Per-feature CV across 54 technical replicate slides is plotted against mean log_2(MFU). Each point represents one peptide-isotype probe (n=187 detected probes after detection filtering); CV was computed on the linear MFU scale across detected values per feature. LOESS smoothers (span=0.6) with 95% confidence bands show the central trend per isotype. CV decreases monotonically with intensity for both channels, consistent with standard immunoassay behavior in which low-signal features approach the noise floor while high-signal features approach the detection ceiling. IgG and IgM tracks overlay closely above ~log_2(MFU)=10, indicating comparable reproducibility across the two detection channels in the quantitative working range.*

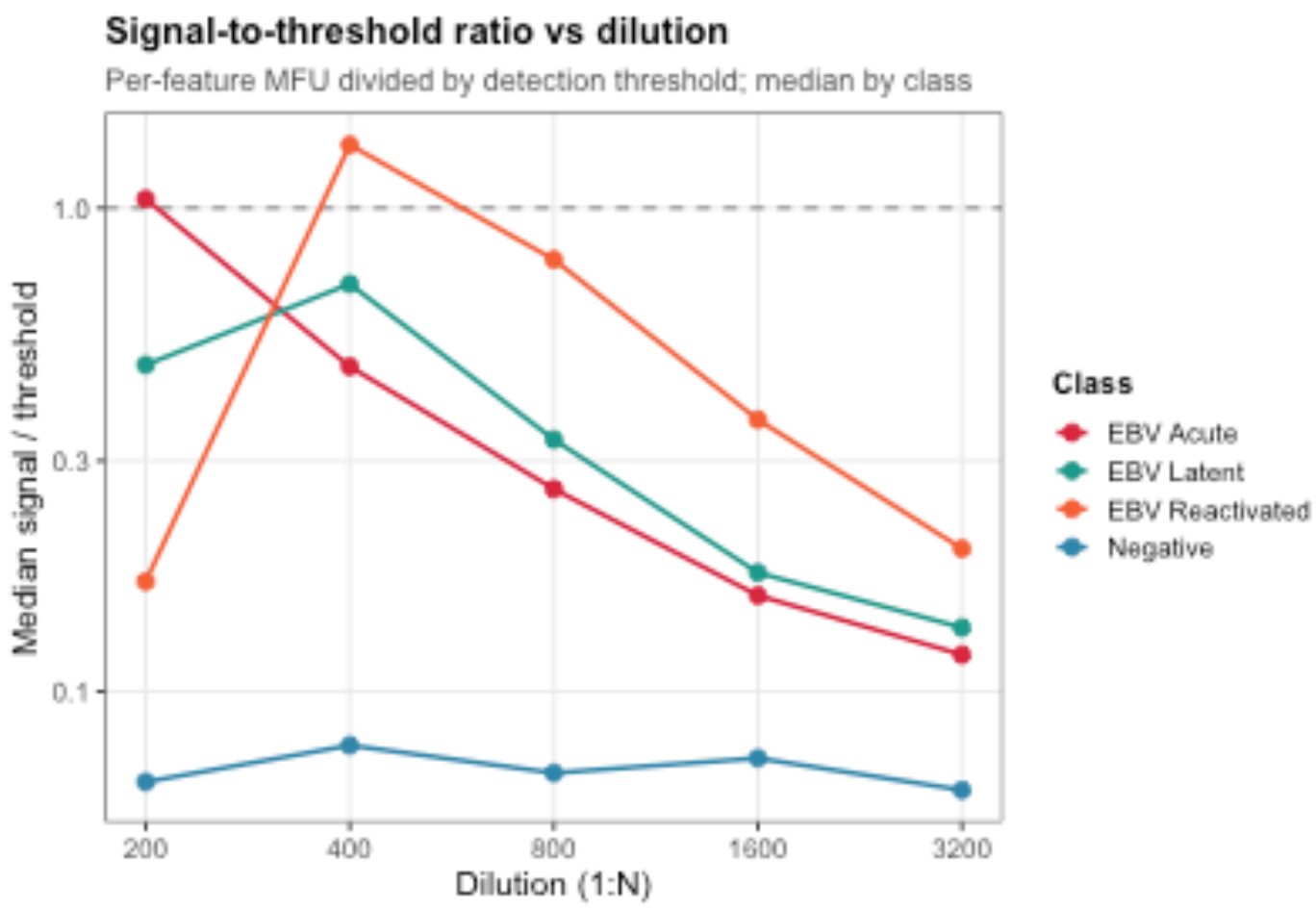

Figure S5. Signal-to-threshold ratio vs. dilution by EBV class. *Median per-feature MFU was divided by a conservative reference threshold (943 MFU), computed within class at each dilution. This reference value was selected a priori as a rigorous 2 SD-above-background working standard; it was chosen rather than calculated and is distinct from the empirical detection threshold. The calculated per-isotype detection thresholds (mean + 2 SD of slide-global background) were 80.9 MFU (IgG) and 170.5 MFU (IgM), as reported in Table S3. EBV Reactivated peaked at 1:400 rather than 1:200, consistent with a high-dose hook effect. EBV Latent showed a similar but smaller pattern. EBV Acute did not show this pattern, potentially reflecting the small Acute cohort and reliance on self-reported clinical history. Larger cohorts with serologically confirmed clinical EBV will be needed to confirm this observation. A 1:400 working dilution was selected to maximize signal while avoiding hook-effect suppression in high-titer samples.*

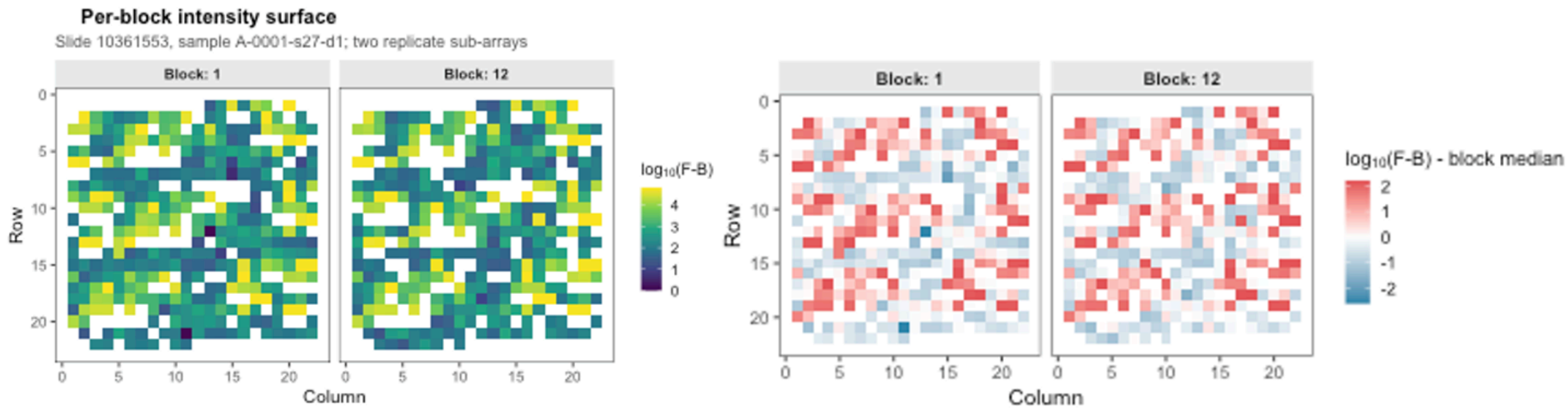

Figure S6. Per-block intensity surface and block-median-centered residuals. *The left panel shows the spatial layout of fluorescence signal for a representative slide/sample (slide 10361553, sample A-0001-s27-d1) printed on two replicate sub-arrays (Block 1 and Block 12). Per-spot $\log_{10}$(F532 Median − B532) intensity rendered on the 22 × 22 print grid shows a mottled pattern, reflecting highly reactive peptides adjacent to non-reactive peptides. The right panel shows the same data after subtracting the block median from each spot, isolating positional deviation from the central tendency. A spatial artifact, such as edge dimming, gradient drying, hybridization-chamber meniscus effects, or post-print shadowing, would appear as a coherent regional bias in this view. No systematic spatial structure was evident, and Block 1 and Block 12 showed independent residual patterns. Together, these views indicate that spot-to-spot intensity variation is dominated by peptide sequence rather than position.*

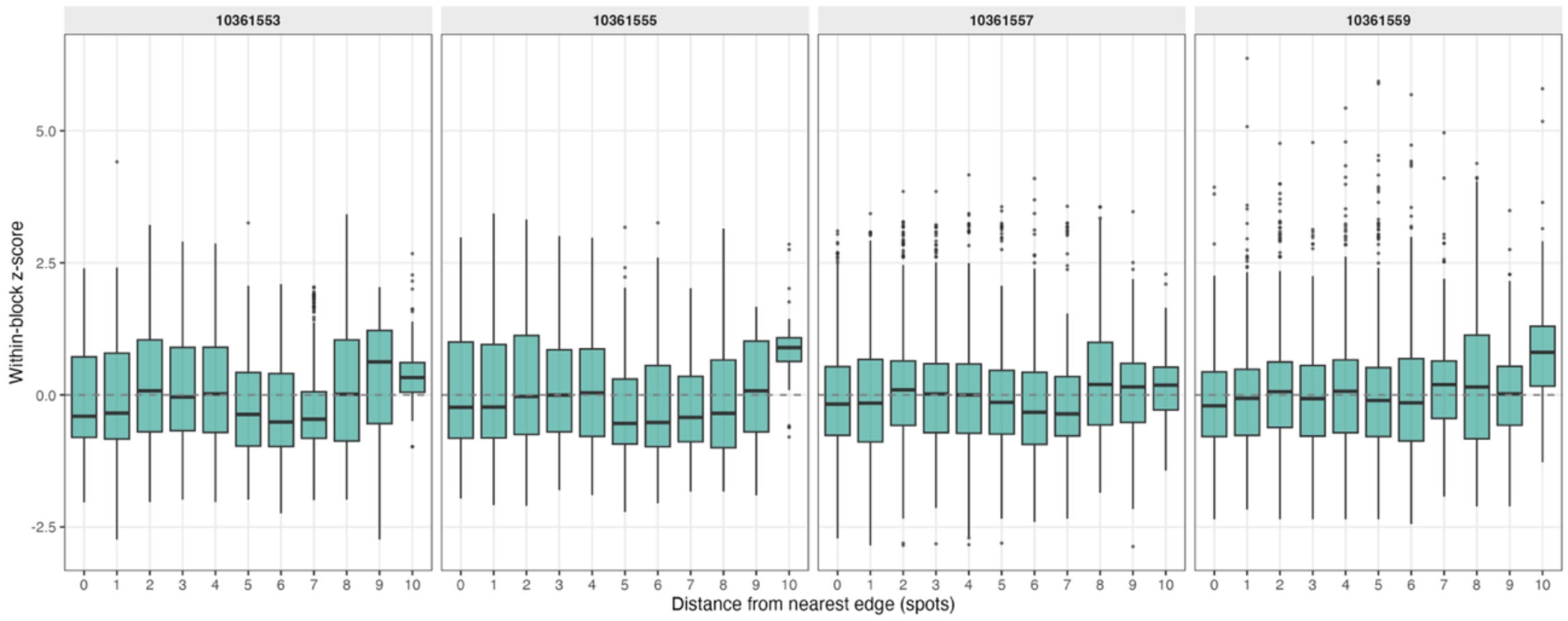

Figure S7. Edge vs. interior spot intensity. *Within-block z-scores are plotted against distance from the nearest sub-array edge (in spot units; 0=edge, 10=block interior) for the four slides in the spatial dataset. Z-scores were computed per block, isolating positional effects from absolute intensity differences between features. Medians were flat across the 0–10 distance range for all four slides, arguing against edge-suppression or edge-concentration artifacts. A modest positive shift (median z≈+0.3 to +0.6) was reproducible at distance 10, the center of the 22 × 22 sub-array, in three of four slides. The magnitude was below the within-block CV (median 14.1%) and was not present in every slide, but it remains a target for ongoing optimization. Candidate improvements include peptide arrangement and packing, distribution of peptide replicates relative to the slide gasket and to each other, peptide concentration in the spotting solution, and spotting buffer composition. Refinement of these conditions is expected to reduce this signal in subsequent platform versions.*

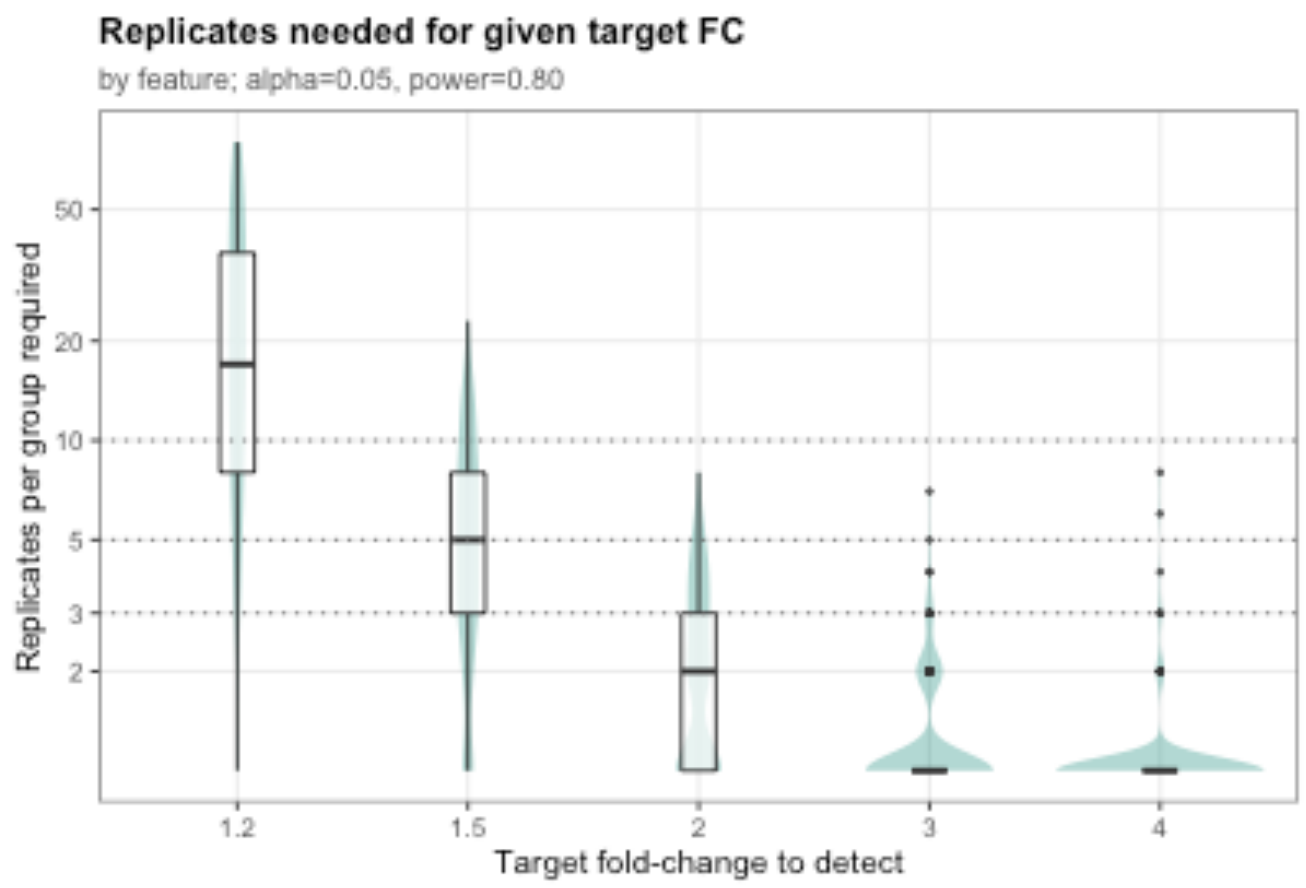

Figure S8. Empirical minimum detectable fold-change. *Distribution of |log_2 ratio| values was computed across all pairs of technical replicate slides for each of the 59 detected features (≥80% above-threshold detection across the 54 slides), pooled across features (n=75,410 replicate pairs). The x-axis shows |log_2 ratio| on the lower scale and the equivalent linear fold-change on the upper scale. The dashed line indicates the 95th percentile, corresponding to an empirical minimum detectable fold-change of approximately 2-fold across replicate-slide pairs. This value represents the smallest fold-difference between two single-slide measurements that can be reliably distinguished from technical noise alone, without distributional assumptions. The empirical estimate and a conventional parametric power analysis at N=3 replicates were concordant at approximately 2-fold.*

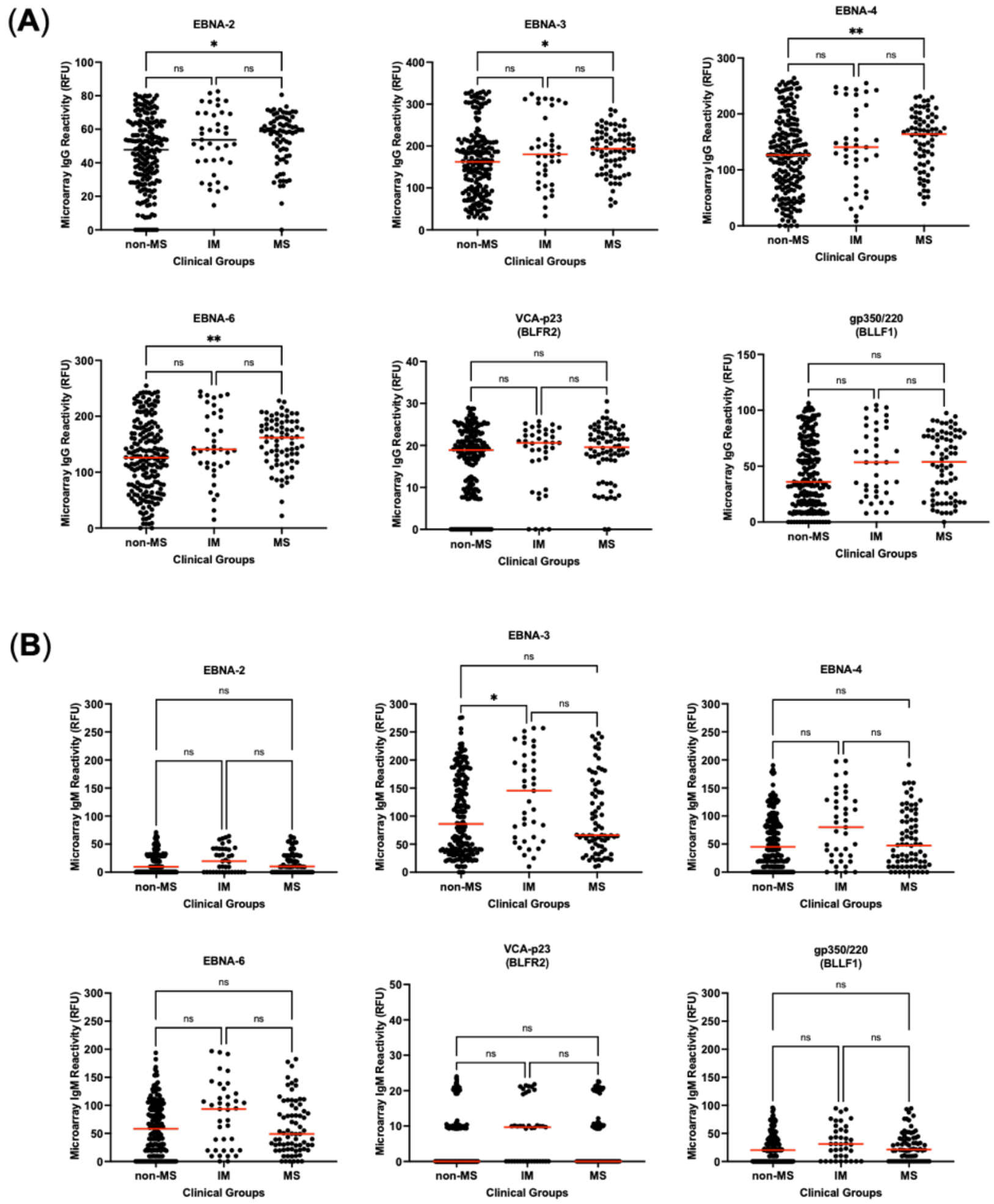

Figure S9. Comparative reactivity to EBV antigens across clinical groups. *Box plots show **(A)** IgG and **(B)** IgM responses to EBNA-2–6, VCA-p23 (BLRF2), and gp350/220 (BLLF1) across non-MS, infectious mononucleosis (IM), multiple sclerosis (MS), and comparator cohorts (non-CRC and CRC). These extended antigen-level comparisons complement the main figures by showing cohort-associated patterns. Statistical significance was assessed using Kruskal–Wallis testing with Dunn's multiple comparisons (*p<0.05, **p<0.005, ****p<0.0001).*
